# From Algorithmic Performance to Clinical Translation: Translational Readiness of Imaging-Based Artificial Intelligence in Dentistry—A Systematic Review

**DOI:** 10.3390/healthcare14131952

**Published:** 2026-07-01

**Authors:** Carlos M. Ardila, Anny M. Vivares-Builes, Eliana Pineda-Vélez

**Affiliations:** 1 Department of Periodontics, Saveetha Dental College and Hospitals, Saveetha Institute of Medical and Technical Sciences, Saveetha University, Chennai 600077, India; 2 Biomedical Stomatology Research Group, Basic Sciences Department, Faculty of Dentistry, Universidad de Antioquia U de A, Medellín 050010, Colombia; anny.vivares@uam.edu.co (A.M.V.-B.); eliana.pineda@uam.edu.co (E.P.-V.); 3 Faculty of Dentistry, Institución Universitaria Visión de las Américas, Medellín 050040, Colombia

**Keywords:** artificial intelligence, dentistry, external validation, generalizability, federated learning

## Abstract

Background/Objectives: Artificial intelligence is increasingly applied to dental imaging, yet favorable internal performance does not necessarily indicate clinical transferability. This systematic review evaluated whether imaging-based dental artificial intelligence models have progressed beyond internal algorithmic development toward external validation, generalizability, reproducibility, privacy-preserving learning, and clinical implementation readiness. Methods: Searches were conducted in PubMed/MEDLINE, Scopus, and Embase up to May 2026. Eligible studies were primary empirical investigations based on human dental or oral imaging data that assessed at least one translational-validation dimension beyond internal development, including external testing, multicenter or multi-device validation, cross-dataset reproducibility, or privacy-preserving learning. Evidence was synthesized using a structured narrative synthesis reported according to the Synthesis Without Meta-analysis framework. Results: Fifteen studies published between 2023 and 2026 were included. They addressed caries detection, periodontal bone loss, gingival inflammation, root morphology, palatal radicular grooves, radiographic quality control, tooth-width estimation, and dental-structure segmentation. Translational-readiness domains included external validation, generalizability, reproducibility, privacy-preserving learning, transparency, and workflow relevance. Validation varied across cohorts, repositories, centers, devices, cross-dataset benchmarks, and federated-learning settings. Reproducibility, annotation harmonization, uncertainty reporting, explainability, workflow evaluation, and code or model availability were inconsistent. Quantitative pooling was not performed because tasks, modalities, units of analysis, reference standards, validation designs, and metrics were highly heterogeneous. Conclusions: Within this selected subset of externally tested studies, translational progress is emerging but remains uneven. Implementation readiness requires stronger reproducibility, clinically meaningful validation, workflow evaluation, and attention to regulatory, organizational, and human-factor barriers.

## 1. Introduction

Artificial intelligence (AI) and machine learning have rapidly expanded across dentistry, particularly in image-based diagnosis, segmentation, treatment planning, risk prediction, and digital workflow support. This growth is consistent with the broader movement toward data-driven healthcare, where computational models are expected to assist clinicians, reduce repetitive tasks, and improve the consistency of image interpretation. In dentistry, these expectations are especially visible because many clinical decisions depend on radiographs, cone–beam computed tomography (CBCT), intraoral photographs, three-dimensional scans, and other structured imaging outputs.

Broad scoping evidence has shown that dental machine-learning research includes a wide spectrum of tasks, input data types, model architectures, reference standards, and performance metrics, with classification, detection, and segmentation being among the most frequent applications [1,2]. However, this same diversity has also generated an evidence base that is difficult to compare, reproduce, and translate into clinical practice.

Most dental AI studies have been designed to demonstrate that a model can learn from a development dataset and achieve favorable internal performance. Such evidence is important, but it does not establish whether a model will remain accurate when exposed to patients, devices, centers, acquisition protocols, disease distributions, or annotation standards that differ from those used during model training. Generalizability is therefore a central condition for clinical implementation. Earlier mapping of machine-learning studies in dentistry found that external validation was uncommon and that many models relied on single-center datasets, internal train–test partitions, or cross-validation without evaluating performance in genuinely independent data [1]. More recent field-wide evidence has continued to identify limited assessment of bias, outliers, calibration, reproducibility, and data access as persistent barriers to responsible adoption [2]. These limitations are not merely technical details; they affect whether a model can be trusted outside the environment in which it was developed.

The problem is also evident in disease-specific reviews. In caries detection, a systematic review and meta-analysis identified 45 studies using AI platforms on dental radiographs or clinical images and reported high heterogeneity in performance, dataset size, caries definitions, annotation procedures, and reporting quality. Although the pooled diagnostic estimates suggested promising performance, the review also found substantial variation across imaging modalities and reported that none of the included studies validated their model on external data according to the Checklist for Artificial Intelligence in Medical Imaging (CLAIM) assessment [3]. This is an important distinction for the present review. The question is not only whether AI can detect caries or segment dental structures with high internal accuracy, but whether those results are transportable across external settings and sufficiently reproducible to support clinical decision-making.

Concerns about interpretability, bias, and generalizability have been addressed more directly in recent systematic evidence. A review focused on explainability, bias, and generalizability included 11 studies and concluded that trustworthiness attributes are relevant to dental AI, but that the included literature remained heterogeneous across dental domains, model types, and outcome definitions [4]. That review is close to the present topic, but its emphasis was on model interpretability and equity rather than on the methodological pathway from internal performance to external clinical translation. The current review is therefore positioned differently. It will focus specifically on studies that go beyond internal validation by empirically evaluating external validation, multicenter or multi-device generalization, cross-dataset reproducibility, privacy-preserving or federated learning, and robustness to data heterogeneity.

This distinction is important because implementation-ready AI requires more than a high area under the curve, Dice similarity coefficient (DSC), or accuracy value obtained in a familiar dataset. Dental imaging data are highly heterogeneous. Differences in radiographic devices, CBCT systems, intraoral scanners, image resolution, acquisition settings, patient age, dentition status, restorations, orthodontic appliances, disease prevalence, and annotation protocols can produce domain shift and reduce performance in external datasets. Recent primary studies illustrate this translational gap. Evidence from dental AI beyond imaging has also shown that performance may decline during cross-national external validation, reinforcing the broader concern that internal discrimination is not necessarily preserved when population structure, disease distribution, or data-collection conditions change [5]. Other externally validated models have tested AI systems on independent dental photographs, clinical records, CBCT scans, periapical radiographs, panoramic radiographs, or intraoral scans, showing that generalization can be evaluated but that its strength depends heavily on the independence, diversity, and quality of the external data [6,7,8,9,10,11].

Privacy and data governance create an additional challenge. Dentistry often depends on identifiable or potentially re-identifiable imaging data, because tooth morphology, restorations, and craniofacial structures may act as individualizing features. Direct pooling of multi-institutional dental images may therefore be restricted by ethical, legal, regulatory, and institutional barriers. Federated learning has been proposed as a privacy-preserving framework that allows collaborative model training without directly sharing raw patient data [12]. In dental imaging, empirical studies comparing federated, centralized, and local learning have shown the relevance of this approach for segmentation tasks, while also revealing new challenges related to heterogeneous data quality, labeling inconsistency, noisy clients, computational demands, and model monitoring [13,14]. Thus, privacy-preserving learning is not only a technical alternative to centralized data pooling; it is a key translational safeguard for scalable dental AI.

Reproducibility is another unresolved barrier. Previous reviews have emphasized that dental AI studies often provide incomplete information on data sources, preprocessing, data partitioning, model specification, calibration, missing data, and external validation, limiting replication and benchmarking [1,2]. Open datasets, public code, transparent reporting of train–validation–test structures, clear reference standards, and cross-dataset evaluations are essential for determining whether a model is robust or merely optimized for a specific dataset. This issue is particularly relevant for segmentation and measurement tasks, where performance can be affected by annotation conventions, unit of analysis, and whether metrics are calculated at the pixel, tooth, image, or patient level. Without reproducible reporting, apparently strong performance may not translate into clinically reliable implementation.

In this review, translational readiness refers to the extent to which an imaging-based dental AI model has been evaluated beyond internal algorithmic performance and provides evidence relevant to clinical transferability. This concept was operationalized across three related levels. Technical validation refers to model performance in development or test datasets, including task-specific metrics such as accuracy, area under the curve, Dice similarity coefficient, or measurement error. Clinical validation refers to evaluation in independent patients, datasets, centers, devices, acquisition protocols, or clinical reference standards that differ from the development setting. Implementation readiness refers to evidence that the model can be reproduced, integrated into clinical workflows, interpreted by users, monitored safely, and used under appropriate regulatory, ethical, and governance conditions. This distinction is important because strong technical performance does not necessarily imply clinical validity or readiness for routine implementation.

Against this background, the present systematic review does not seek to re-estimate the overall diagnostic accuracy of AI in dentistry. Instead, it aims to synthesize the subset of imaging-based dental AI studies that directly address translational readiness through external validation, independent dataset testing, multicenter or multi-device generalizability, cross-dataset reproducibility, privacy-preserving or federated learning, explainability, reporting transparency, and robustness to data heterogeneity.

Before conducting this review, we expected that only a small and methodologically heterogeneous subset of dental AI studies would have progressed beyond internal validation. This expectation was based on previous evidence showing that many dental AI models are developed and tested within limited datasets, with incomplete assessment of external validity, reproducibility, calibration, transparency, and clinical workflow relevance. We conducted this review to clarify whether recent imaging-based dental AI studies have begun to address these translational gaps and to identify which validation designs provide stronger evidence of clinical transferability. The novelty of this review lies in shifting the focus from algorithmic performance alone to the translational pathway from external validation and generalizability to reproducibility, privacy-preserving learning, and implementation readiness.

Evidence was organized through a structured narrative synthesis reported in accordance with the Synthesis Without Meta-analysis (SWiM) framework. Studies were grouped into predefined domains related to clinical transferability and implementation readiness, including external validation, generalizability, reproducibility, privacy-preserving learning, transparency, and clinical workflow relevance. This approach was selected because the review focuses on the translational validation of imaging-based dental AI rather than on a single diagnostic comparison or intervention effect.

The primary objective is to evaluate the extent to which dental AI models have moved beyond internal algorithmic performance toward reproducible, externally validated, and clinically transferable implementation.

## 2. Materials and Methods

This systematic review was conducted in accordance with the Preferred Reporting Items for Systematic Reviews and Meta-Analyses (PRISMA) 2020 statement [15]. The protocol was prospectively registered in the International Prospective Register of Systematic Reviews (PROSPERO; CRD420261402398). The search strategy and reporting of information sources were planned to support transparency and reproducibility, in line with PRISMA-S recommendations for systematic review searches [16].

### 2.1. Eligibility Criteria

Eligibility criteria were defined using an AI-validation review framework rather than a conventional intervention PICO. This decision was made because the objective of the review was not to compare a single AI intervention with a single clinical comparator, but to evaluate whether imaging-based dental AI studies have progressed beyond internal algorithmic performance toward external validation, generalizability, reproducibility, privacy-preserving learning, and clinical implementation readiness.

Population/data source: Eligible studies used human dental, oral, craniofacial, or maxillofacial imaging data. These data included dental radiographs, panoramic radiographs, periapical or bitewing radiographs, cone–beam computed tomography scans, intraoral photographs, dental photographs, intraoral scans, three-dimensional dental models, or other imaging-based oral-health datasets.

Index AI approach: The index approach was an artificial intelligence or machine-learning model applied to an imaging-based dental task. Eligible models included machine learning, deep learning, convolutional neural networks, transformer-based models, segmentation models, radiomics, federated-learning models, multimodal AI, or related computational approaches.

Target task: Eligible tasks included dental diagnosis, detection, classification, segmentation, measurement, radiographic quality control, risk prediction, or decision support. Studies were not required to evaluate the same clinical condition, because the review question focused on translational validation rather than on one diagnostic indication.

Validation setting: To be eligible, studies had to include empirical evidence beyond internal model development. This included at least one of the following: independent external testing, patient-level external validation, multicenter validation, multi-device or multi-acquisition testing, cross-dataset generalizability, federated or privacy-preserving learning, robustness to dataset heterogeneity, or reproducibility assessment. Studies limited only to internal train–validation–test splitting, k-fold cross-validation, or internal hold-out testing were not eligible unless they also included one of these external or translational validation components.

Reference standard or comparator: Reference standards and comparators were recorded according to the design and task of each study. They could include expert or consensus annotation, manual or refined segmentation, cone–beam computed tomography confirmation, longitudinal clinical documentation, internal validation results, centralized or local learning, or clinician assessment. These elements were not treated as a single common comparator because they served different methodological functions across diagnostic, segmentation, measurement, and federated-learning studies.

Outcomes: The primary outcomes were external-validation performance, generalizability across datasets, centers, devices, acquisition protocols, populations, or annotation settings, cross-dataset reproducibility, privacy-preserving or federated-learning performance, internal-to-external performance change when directly comparable, and methodological indicators of translational readiness. Task-specific quantitative metrics, including AUC, accuracy, sensitivity, specificity, precision, recall, F1-score, negative predictive value, Dice similarity coefficient, intersection over union, Hausdorff distance, HD95, average symmetric surface distance, root mean square error, mean absolute error, and other reported performance measures, were extracted according to the task evaluated in each study.

Study design: Eligible designs included primary empirical studies, diagnostic accuracy studies with external validation, model-development studies with independent validation, segmentation validation studies, multicenter or multi-device validation studies, cross-dataset studies, federated-learning studies, and retrospective or prospective validation studies. Studies comparing AI performance with expert clinicians or established reference standards were eligible when they also contributed evidence relevant to external validation, generalizability, reproducibility, or clinical transferability.

### 2.2. Exclusion Criteria

Studies were excluded when they were systematic reviews, scoping reviews, narrative reviews, umbrella reviews, editorials, letters, commentaries, protocols, dissertations, or conference abstracts without sufficient methodological and outcome data. Studies were also excluded when they used animal data only, purely simulated datasets without clinical or human-derived dental data, non-dental medical datasets, or broader medical AI datasets from which dental-specific results could not be separated.

Purely technical algorithm papers were excluded when they did not include empirical validation in dental data. Studies limited to internal train–validation–test partitioning, k-fold cross-validation, or internal hold-out testing were excluded unless they also included an external dataset, independent cohort, multicenter or multi-device evaluation, cross-dataset testing, federated or collaborative learning comparison, or reproducibility/generalizability assessment. Studies assessing non-AI digital tools, conventional statistical models without an AI or machine-learning component, or manual-only diagnostic methods were not eligible.

### 2.3. Information Sources and Search Strategy

A comprehensive literature search was performed in PubMed/MEDLINE, Scopus, and Embase. Searches were conducted without language restrictions and included articles published up to May 2026, with no restriction on the initial publication date. Additional records were identified through backward reference checking, forward citation tracking, and screening of relevant reviews. Authors were contacted when essential methodological or performance information was unavailable in the published report.

The search strategy combined controlled vocabulary and free-text terms related to AI, machine learning, deep learning, federated learning, dentistry, dental imaging, external validation, generalizability, reproducibility, independent datasets, multicenter testing, privacy-preserving learning, and cross-dataset evaluation. The complete search strategy for each database is provided in Supplementary Table S1.

### 2.4. Selection Process

All records retrieved from the databases were imported into reference-management software, and duplicates were removed before screening. Two reviewers independently screened titles and abstracts according to the eligibility criteria. Full texts of potentially eligible articles were then retrieved and assessed independently by the same reviewers. Disagreements were resolved through discussion, and a third reviewer was consulted when consensus was not reached. Reasons for exclusion at the full-text stage were recorded and summarized in Supplementary Table S2.

### 2.5. Data Collection Process

Data extraction was performed independently by two reviewers using a prepiloted extraction form designed for this review. Extracted information was compared between reviewers, and discrepancies were resolved through discussion. When necessary, authors were contacted to clarify missing information related to external validation, dataset independence, model performance, or availability of code, models, or data.

### 2.6. Data Items

The following variables were extracted from each included study: bibliographic information, country, dental specialty or application, study design, data source, clinical setting, sample size, unit of analysis, imaging modality, AI model type, clinical or technical task, training strategy, validation strategy, external-validation design, reference standard, comparator, internal performance metrics, external performance metrics, uncertainty estimates, availability of code, model, or dataset, explainability methods, privacy-preserving methods, demographic or device heterogeneity, evidence of data leakage prevention, and the main translational limitations reported by the authors.

External validation was categorized according to the most specific level reported in each study: patient-level external validation, independent image-dataset validation, multicenter validation, multi-device or multi-acquisition validation, cross-dataset generalizability testing, or federated/collaborative learning across distributed data sources. For studies reporting both internal and external performance, the internal-to-external performance change was extracted or calculated when the same metric was available in both settings.

### 2.7. Study Risk of Bias and Reporting Quality Assessment

Risk of bias was assessed according to the methodological design and AI task of each study. For diagnostic accuracy, classification, and detection studies, the Quality Assessment of Diagnostic Accuracy Studies 2 (QUADAS-2) tool was used [17]. The assessment considered patient selection, conduct and interpretation of the index AI test, appropriateness of the reference standard, and flow and timing. AI-specific issues were also considered, including independence of the external dataset, possible data leakage, threshold selection, blinding to the reference standard, and applicability of the validation sample to the review question.

For studies based on clinical or epidemiological prediction models, the Prediction model Risk of Bias ASsessment Tool (PROBAST) was used when applicable [18]. The domains included participants, predictors, outcome, and analysis, with attention to overfitting, model calibration, handling of missing data, and adequacy of the validation strategy.

For segmentation, measurement, cross-dataset, and federated-learning studies where QUADAS-2 or PROBAST did not fully capture the relevant sources of bias, an adapted AI-specific methodological appraisal was applied. This appraisal was not intended to generate a separate pooled quality score. Instead, it was used to identify AI-specific threats to validity and implementation relevance that were not fully captured by conventional diagnostic or prediction-model tools. The seven domains assessed whether the data sources and validation datasets were independent, whether the annotation or reference standard was clearly defined and reproducible, whether leakage prevention was described, whether the validation design tested transportability beyond the development setting, whether statistical uncertainty was reported, whether code, models, datasets, or preprocessing steps were sufficiently transparent for reproducibility, and whether the evaluation was clinically applicable to the intended dental workflow. This appraisal assessed seven domains: data source and independence; annotation or reference-standard quality; prevention of data leakage; external validation and transportability; statistical analysis and uncertainty reporting; reproducibility and transparency, including code, model, and data availability; and clinical applicability. Reporting transparency was additionally examined using CLAIM-derived items, particularly for imaging-based AI studies [19]. Risk-of-bias and reporting-quality assessments were conducted independently by two reviewers and summarized in tabular form.

### 2.8. Certainty of Evidence Assessment

The certainty of evidence was evaluated using a Grading of Recommendations Assessment, Development and Evaluation (GRADE)-informed framework [20]. Because the included studies differed substantially in task, modality, unit of analysis, validation design, and performance metric, certainty was not assigned to a single global outcome. Instead, certainty was summarized by functional domain. The domains considered were risk of bias, inconsistency, indirectness, imprecision, and publication or reporting bias. Certainty was rated as high, moderate, low, or very low, with downgrading decisions justified narratively for each domain. For domains without quantitative pooling, certainty judgments were based on the consistency of findings, independence of external validation, transparency of reporting, and clinical applicability of the validation setting.

### 2.9. Data Synthesis and Statistical Analysis

The primary synthesis was planned as a structured narrative synthesis reported according to the Synthesis Without Meta-analysis (SWiM) framework, because the review addressed a translational AI-validation question rather than a single diagnostic or intervention outcome. The review was not designed to estimate whether AI is more accurate than dentists for one diagnostic condition. Instead, it was designed to evaluate whether imaging-based dental AI models had been assessed beyond internal development through distinct but related indicators of clinical transferability, including external validation in data not used for model development, multicenter or multi-device generalizability, cross-dataset reproducibility, privacy-preserving learning, and other implementation-readiness dimensions.

The synthesis followed a reproducible multistep process. First, a study-by-domain matrix was created to map each included study to one or more translational-readiness domains: external validation and independent test cohorts; multicenter, multi-device, or cross-domain generalizability; cross-dataset reproducibility and annotation heterogeneity; privacy-preserving and federated learning; explainability, transparency, and reproducibility; and clinical workflow relevance or implementation readiness. Second, each study was coded for validation type, unit of analysis, image modality, task, reference standard, comparator, internal performance, external performance, and availability of reproducibility resources. Third, external validation was classified as patient-level, dataset-level, center-level, device-level, cross-dataset, or federated/distributed validation. These validation categories were not interpreted as equivalent levels of evidence. For the purposes of synthesis, multicenter, multi-device, cross-dataset, and federated/distributed validation were considered to provide stronger evidence of transportability because they tested model performance under broader sources of domain shift. By contrast, validation in a single independent cohort or image repository was interpreted as evidence of external testing, but not necessarily as evidence of broad clinical generalizability when center diversity, device heterogeneity, acquisition protocols, population characteristics, or annotation harmonization were limited or unclear. Fourth, studies were compared within each functional domain to identify recurring methodological strengths, translational limitations, and possible sources of performance degradation. Fifth, the domain-level findings were integrated into a structured narrative that distinguished evidence of clinical transferability from evidence limited to algorithmic performance in familiar datasets. This synthesis was reported according to the SWiM framework because quantitative pooling was inappropriate [21].

Exploratory quantitative synthesis was planned only if at least three studies reported sufficiently comparable metrics within the same task family, validation context, unit of analysis, and outcome metric. Feasibility assessment for quantitative synthesis considered not only the number of available studies but also comparability in clinical task, imaging modality, anatomical target, unit of analysis, reference standard, validation design, and outcome metric. Quantitative pooling was considered inappropriate when these elements differed sufficiently to compromise the interpretability of a pooled estimate. Candidate analyses included pooled AUC for comparable diagnostic or classification studies with external validation, pooled Dice similarity coefficient for comparable segmentation studies with external validation or cross-dataset testing, and pooled internal-to-external performance change when the same metric was reported in both internal and external settings within the same study.

Quantitative pooling was not planned across all included studies because the review was expected to include heterogeneous AI tasks, imaging modalities, reference standards, validation designs, units of analysis, and metric families. A global diagnostic meta-analysis would only be performed if the included studies provided a clinically and statistically coherent set of comparable outcomes. Forest plots were planned only for outcome groups that met the criteria for exploratory quantitative synthesis.

If quantitative synthesis was feasible, random-effects models would be used because clinical and methodological heterogeneity was expected. Restricted maximum likelihood estimation would be preferred for between-study variance, and results would be reported with 95% confidence intervals. Heterogeneity would be evaluated using visual inspection of forest plots, the I^2^ statistic, and tau-squared (τ^2^) [22]. Studies without adequate uncertainty estimates would be retained in the structured narrative synthesis but would not be included in quantitative pooling unless sufficient data could be derived from available information. Analyses were planned in R 4.3.2 (R Foundation for Statistical Computing, Vienna, Austria) using the metafor package [23].

## 3. Results

### 3.1. Study Selection

The database search identified 1308 records, including 212 records from PubMed/MEDLINE, 651 from Scopus, and 445 from Embase. After removing 385 duplicate records, 923 unique records were screened by title and abstract. Most records were excluded at this stage because they did not address artificial intelligence models in dentistry or oral healthcare, were not based on human dental or oral imaging data, focused on non-dental medical applications, were reviews, editorials, protocols, or conference abstracts, or reported only internal model development without evidence of external validation, independent dataset testing, multicenter assessment, cross-dataset evaluation, federated learning, or reproducibility analysis.

After title and abstract screening, 24 articles were considered potentially eligible and were assessed in full text. Nine articles were excluded after full-text evaluation. The most frequent reasons were restriction to internal train–validation–test performance, absence of an independent external dataset or cross-setting evaluation, lack of empirical evidence related to generalizability, privacy-preserving learning, or reproducibility, or use of non-imaging data outside the final imaging-based scope of the review. The excluded full-text articles and reasons for exclusion are presented in Supplementary Table S2.

**Figure 1:**
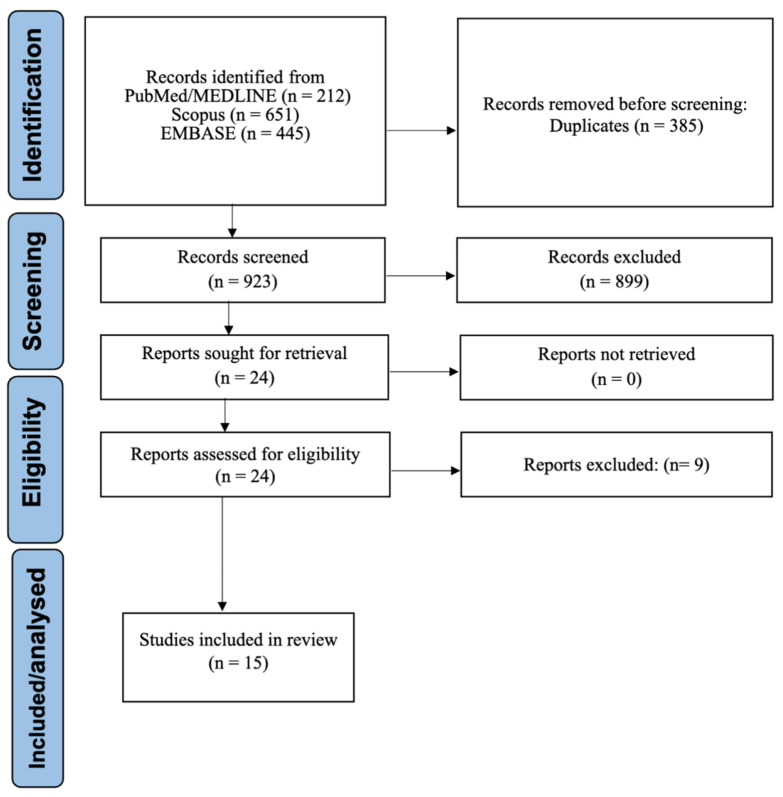
PRISMA flow diagram of study identification, screening, full-text assessment, and inclusion.

Overall, 15 studies met all inclusion criteria and were included in the systematic review [6,7,8,9,10,11,13,14,24,25,26,27,28,29,30]. These studies formed the evidence base for evaluating external validation, generalizability, cross-dataset reproducibility, privacy-preserving or federated learning, and translational readiness of imaging-based artificial intelligence models in dentistry. The study selection process is summarized in Figure 1.

After full-text assessment and data extraction, the feasibility of quantitative synthesis was evaluated for the predefined candidate outcome groups described in the protocol. Detection and classification studies most commonly reported AUC-based metrics, whereas segmentation studies primarily reported Dice similarity coefficient (DSC), intersection over union (IoU), or Hausdorff-distance measures. However, no outcome group contained at least three studies that were sufficiently comparable with respect to clinical task, imaging modality, anatomical target, unit of analysis, reference standard, validation setting, and reported performance metric. For example, AUC values were reported across fundamentally different applications, including caries detection, root-number classification, radiographic quality assessment, and palatal radicular groove diagnosis, while DSC values were reported for segmentation tasks involving different anatomical structures, imaging modalities, and validation frameworks. In addition, uncertainty estimates and reporting formats were inconsistent across studies. Therefore, quantitative pooling and forest plots were not considered methodologically appropriate, and the evidence was synthesized through a structured narrative synthesis organized according to predefined translational-readiness domains.

### 3.2. General Characteristics of the Included Studies

The 15 included studies were published between 2023 and 2026, with most appearing in 2025 or 2026 [6,7,8,9,10,11,13,14,24,25,26,27,28,29,30]. The corpus covered a broad range of imaging-based dental AI applications rather than a single diagnostic task. These included caries detection and classification [10,24,25], caries and periodontal bone loss assessment using commercial clinical decision-support systems [6], gingival inflammation grading [29], root-number detection in maxillary premolars [11], palatal radicular groove diagnosis and classification in CBCT scans [7], radiographic quality control in periapical radiographs [8], tooth-width estimation from standardized occlusal photographs [30], and automated segmentation of teeth, pulp, gingiva, or dental hard tissues across panoramic radiographs, CBCT scans, intraoral scans, and three-dimensional dental models [9,13,14,26,27,28].

The included studies also differed in how they tested model transportability. Some evaluated independent external datasets or patient-level external validation [6,10,11,24,25,30], whereas others assessed multicenter, multi-device, cross-dataset, or federated-learning settings [7,8,9,13,14,26,27,28,29]. This variation was central to the review question: the included evidence did not support a single pooled diagnostic accuracy framework, but it did allow a structured assessment of how dental AI models behave when tested beyond their original development environment. Detailed study characteristics, including dental domain, imaging modality, AI task, sample size, unit of analysis, model type, reference standard, and validation approach, are presented in Table 1.

### 3.3. AI Tasks, Imaging Modalities, and Reference Standards

The 15 included studies covered a heterogeneous but clearly image-centered set of artificial intelligence applications in dentistry [6,7,8,9,10,11,13,14,24,25,26,27,28,29,30]. At the level of primary task, five studies focused mainly on segmentation [9,13,14,26,28], five on detection, classification, or grading tasks [6,8,11,24,29], four combined detection or classification with segmentation outputs [7,10,25,27], and one addressed quantitative measurement of dental morphology [30]. This distribution shows that the included literature extends beyond conventional lesion detection and also includes structurally oriented tasks such as tooth segmentation, quality control, and morphometric estimation.

**Figure 2:**
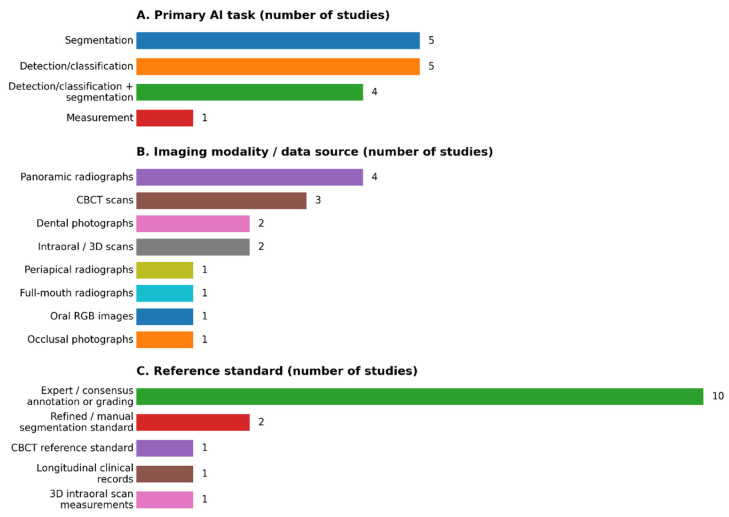
AI, artificial intelligence; CBCT, cone–beam computed tomography; RGB, red–green–blue. Distribution of artificial intelligence tasks, imaging modalities, and reference standards across the included studies. The figure summarizes the distribution of the 15 included studies according to three descriptive dimensions: primary artificial intelligence task, dominant imaging modality or data source, and principal reference standard. Task categories were assigned according to the main objective of each study. Imaging modality was classified according to the primary data source used for model development or external validation. Reference-standard categories reflect the main method used to define ground truth, including expert or consensus annotation, refined or manual segmentation, cone–beam computed tomography-based confirmation, longitudinal clinical documentation, or measurements derived from three-dimensional intraoral scans. Because each study was assigned to one dominant category per dimension, counts within each panel sum to 15.

The imaging sources were similarly diverse. Panoramic radiographs were the most frequent modality [11,13,14,27], followed by CBCT scans [7,9,26]. Other studies used dental photographs [10,24], intraoral or three-dimensional scans [25,28], periapical radiographs [8], full-mouth radiographic series linked to clinical follow-up [6], standard oral RGB images [29], or standardized occlusal photographs [30]. Reference standards were predominantly based on expert or consensus annotation and grading procedures [7,8,10,13,14,24,25,27,28,29], although some studies relied on refined or manual segmentation standards [9,26], CBCT-confirmed morphology [11], longitudinal clinical documentation [6], or mesiodistal measurements derived from three-dimensional intraoral scans [30]. Taken together, these distributions highlight the methodological heterogeneity of the included evidence and reinforce the rationale for a structured narrative synthesis rather than a single pooled diagnostic-accuracy framework. This heterogeneity also affected interpretability. Performance estimates could not be interpreted as reflecting a common clinical effect because studies differed not only in numerical metrics but also in diagnostic task, anatomical target, imaging modality, reference standard, validation design, and unit of analysis. Therefore, higher performance in one study did not necessarily indicate greater clinical transferability than lower performance in another study evaluating a more complex task or broader validation setting. A summary of the distribution of AI tasks, imaging modalities, and reference standards is presented in Figure 2.

### 3.4. External Validation and Independent Dataset Testing

The included studies did not apply a single model of external validation. Instead, they represented several levels of independence, ranging from independent image datasets and patient-level external cohorts to multicenter, multi-device, cross-dataset, and federated-learning validation frameworks. This distinction was important because a model tested on new images from the same workflow does not face the same translational challenge as a model evaluated across different centers, scanners, annotation protocols, or distributed data silos.

Independent external validation was most explicit in studies that used separate patient cohorts, image repositories, or external test sets not involved in model development [6,10,11,24,25,30]. Other studies evaluated transportability through multicenter or multi-device settings, including cone–beam computed tomography (CBCT) scans from different systems, periapical radiographs from different acquisition technologies, and panoramic radiographs from multiple international centers [7,8,9,13,14,26,27,28,29]. Cross-dataset testing was especially informative when annotation heterogeneity itself became part of the validation problem, as shown in the AKUDENTAL study [27]. Federated-learning studies contributed a different form of external validity by evaluating whether models trained across distributed sources could improve generalizability without direct data sharing [13,14].

These validation frameworks were not interpreted as equivalent forms of translational evidence. Studies incorporating multicenter, multi-device, cross-dataset, or federated/distributed validation were considered to provide stronger evidence of transportability because they evaluated model performance under broader forms of domain shift. In contrast, studies based on a single independent patient cohort, external image repository, or external test set were interpreted as demonstrating external testing but providing more limited evidence of generalizability when diversity of centers, devices, acquisition protocols, populations, or annotation practices was restricted. Consequently, greater interpretive weight was assigned to studies that evaluated model performance across multiple sources of heterogeneity rather than within a single external dataset.

Across studies, the external metrics were task-specific rather than directly interchangeable. Classification and detection studies generally reported accuracy, sensitivity, specificity, area under the curve (AUC), concordance, negative predictive value (NPV), precision, recall, F1-score, or mean average precision (mAP), whereas segmentation and measurement studies used Dice similarity coefficient (DSC), intersection over union (IoU), 95th percentile Hausdorff distance (HD95), mean intersection over union (mIoU), mean absolute error (MAE), root mean square error (RMSE), or agreement statistics. Several studies reported some degree of internal-to-external performance change, but the form of this comparison varied substantially. For this reason, the external-validation evidence was synthesized functionally rather than treated as a single diagnostic-accuracy outcome. The external-validation characteristics of the included studies are summarized in Table 2.

### 3.5. Multicenter, Multi-Device, and Cross-Domain Generalizability

Generalizability was evaluated through several validation strategies rather than through a uniform design across studies. Some studies directly tested models across centers, scanners, acquisition systems, or geographically distinct datasets, whereas others assessed generalizability by applying a model to an independent external image set or to a clinical cohort that differed from the development data. This distinction is relevant because transportability in dental AI may be affected not only by patient differences, but also by device type, image acquisition protocol, annotation conventions, dentition status, anatomical complexity, and data-quality variation.

The strongest evidence of multicenter or multi-device generalizability came from studies using CBCT scans from different systems or centers, periapical radiographs acquired with different technologies, panoramic radiographs from multiple institutions, or distributed datasets evaluated under federated-learning conditions [7,8,9,13,14,26,27,28,29]. Other studies contributed more limited but still relevant evidence by testing models on independent image repositories, external patient cohorts, or clinical datasets not used during model development [6,10,11,24,25,30]. Overall, the evidence showed that external performance was often maintained only partially and depended on the degree of similarity between development and validation data. Studies that explicitly examined device heterogeneity, annotation inconsistency, noise, labeling inaccuracy, or dataset shift were particularly informative because they identified why a model may fail or lose performance outside its original training environment. The main generalizability features of the included studies are summarized in Table 3.

### 3.6. Cross-Dataset Reproducibility and Annotation Heterogeneity

Reproducibility was uneven across the included studies. Only a minority provided open or partially accessible resources that would allow independent reanalysis, retraining, or benchmarking. The strongest example was the AKUDENTAL study, which made the dataset and processing, training, and evaluation code available through GitHub and explicitly examined how differences in annotation protocols affected cross-dataset performance [27]. Other studies provided more limited reproducibility resources, such as code with restricted or request-based data access [11], data and code through private or institutional repositories upon request [26], a public web application with data available upon reasonable request [10], or a repository for the analyzed dataset [30]. By contrast, several externally validated studies reported no public release of code, trained models, or patient-level datasets, usually because of privacy restrictions, commercial-system constraints, or the use of institutional imaging data (Table 4).

Annotation heterogeneity was not assessed consistently. In most studies, reference standards were based on expert annotation, consensus diagnosis, refined segmentation, or clinical records, but the reproducibility of those labels across annotators or datasets was rarely the main analytic focus. AKUDENTAL was the clearest exception because it directly showed that cross-dataset performance differences could reflect inconsistent annotation definitions rather than model failure alone [27]. Rubak et al. [14] also addressed this issue experimentally by introducing label manipulation and image-noise scenarios in federated, centralized, and local learning. Chen et al. [29] reported strong agreement among expert annotators for gingival inflammation grading, whereas other studies used consensus or expert adjudication without formal cross-dataset label harmonization. These findings make annotation reproducibility a central translational issue: a model cannot be considered broadly generalizable if the target labels themselves are unstable across datasets, centers, or clinical conventions.

### 3.7. Privacy-Preserving and Federated Learning

Only two included studies directly evaluated privacy-preserving or federated-learning strategies in dental imaging, and both focused on tooth segmentation in panoramic radiographs [13,14]. This limited number was expected because most externally validated dental AI studies still relied on centralized development or independent external testing rather than distributed model training. Nevertheless, these two studies were central to the translational focus of this review because they addressed a problem that conventional external validation cannot solve: how to develop more generalizable dental AI models when raw imaging data cannot be freely pooled across institutions.

The rationale for federated learning is particularly relevant in dentistry. Dental radiographs may contain individualizing anatomical and restorative features, and multi-institutional data sharing can be restricted by privacy, regulatory, and institutional constraints. In this setting, federated learning allows participating centers to train a shared model without exchanging raw patient images; instead, model parameters or learned updates are shared and aggregated centrally [12]. This privacy-preserving logic was empirically tested by Schneider et al. [13], who compared federated learning with local and centralized learning using 4177 panoramic radiographs from nine international centers. Federated learning outperformed local learning in most centers and showed better generalizability on pooled multicenter test data, although centralized learning generally remained superior when direct data pooling was allowed [13]. This finding suggests that federated learning may narrow the gap between isolated local models and centralized models when privacy constraints make direct pooling unrealistic.

Rubak et al. extended this evidence by examining whether federated learning remained robust when data quality and label quality were deliberately compromised [14]. Using 2066 panoramic radiographs from six institutions, the authors compared federated, centralized, and local learning under baseline conditions, label manipulation, image-noise manipulation, and faulty-client exclusion. Federated learning matched or exceeded centralized learning and consistently outperformed local learning across several corruption scenarios, while per-client loss monitoring helped identify corrupted sites [14]. This study was particularly important because it moved beyond the assumption that all participating centers contribute clean and consistently annotated data. In practical multicenter dental AI development, differences in image quality, labeling behavior, and institutional workflow are likely to be common rather than exceptional.

Taken together, these two studies indicate that federated learning is a plausible privacy-preserving route for scalable dental AI development, but not a complete solution to implementation barriers. Its usefulness depends on the quality and balance of local datasets, the reliability of annotations, the ability to monitor anomalous clients, and the technical capacity of participating institutions. The evidence also shows that privacy-preserving learning and generalizability are closely linked: federated learning may improve access to diverse data without direct image sharing, but model performance remains vulnerable to data heterogeneity, labeling inconsistency, and client-level imbalance. Thus, in the current dental AI literature, federated learning should be viewed as an emerging translational strategy rather than a mature deployment standard.

### 3.8. Explainability, Transparency, and Reporting Reproducibility

Explainability and reporting transparency were inconsistently addressed across the included studies. Most articles clearly described the external-validation design and reported the main model architecture, input modality, reference standard, and performance metrics. However, formal explainability methods were uncommon. Only a small number of studies used visual or interpretable outputs to support model interpretation, such as Grad-CAM or attention-related visualizations, whereas most segmentation, measurement, and federated-learning studies focused on external performance, robustness, or reproducibility rather than on explaining individual model decisions.

Transparency was also uneven. Several studies provided enough detail to understand the validation structure, including whether testing was performed on external centers, independent image datasets, cross-dataset benchmarks, or distributed institutional data [6,7,8,9,10,11,13,14,24,25,26,27,28,29,30]. Nevertheless, open reproducibility was limited. Code, datasets, or trained models were publicly available in only a minority of studies, and in some cases access was restricted because of patient privacy, institutional data governance, proprietary software, or commercial AI systems. This pattern is important because a model may appear externally valid within the published study, but independent reproduction remains difficult when data splits, annotations, source code, trained weights, or external datasets are not accessible.

Table 5 summarizes explainability and reporting transparency using CLAIM-derived elements relevant to imaging-based AI studies, including clarity of external validation, reporting of uncertainty, leakage-prevention information, and availability of code, data, or models.

### 3.9. Risk of Bias and Methodological Quality

Risk of bias and methodological quality were assessed according to the methodological design and primary AI task of each study. QUADAS-2 was applied to diagnostic, detection, classification, or grading studies, because these studies evaluated an index test against a reference standard and therefore fitted the diagnostic-accuracy structure of patient or image selection, index-test conduct, reference standard, and flow and timing [17]. PROBAST was retained in the protocol for clinical or epidemiological prediction models [18], but it was not used as a primary appraisal tool in the final synthesis because the final included corpus was restricted to imaging-based studies rather than questionnaire-based or clinical-risk prediction models. For segmentation, measurement, cross-dataset, and federated-learning studies, an adapted AI-specific methodological appraisal was used because conventional diagnostic-accuracy tools do not fully capture issues such as annotation quality, data leakage prevention, external transportability, code and model availability, and distributed-learning robustness.

Across the included studies, the main methodological strengths were the use of external or independent test data, multicenter or multi-device evaluation, and clinically meaningful reference standards. These features reduced the likelihood that model performance reflected only internal optimization. However, important concerns remained. External datasets were often smaller than development datasets, some external validations relied on independent image repositories with limited patient-level metadata, and open reproducibility resources were inconsistently available. For segmentation and measurement studies, the most relevant concerns were not always those captured by standard diagnostic risk-of-bias domains; rather, they involved annotation consistency, availability of manual or refined reference segmentations, reporting of data partitioning, and whether the model could be independently reproduced.

The risk-of-bias and methodological-quality findings should therefore be interpreted as a domain-specific appraisal rather than as a single uniform score across all included studies. In diagnostic or classification studies, the key concerns related to patient or image selection, independence of the external set, and appropriateness of the reference standard. In segmentation, measurement, cross-dataset, and federated-learning studies, the main concerns related to dataset independence, annotation heterogeneity, incomplete open access to code or trained models, and limited reporting of procedures used to prevent data leakage. Table 6 summarizes these judgments by study and appraisal domain.

### 3.10. Structured Synthesis of Translational Readiness

The structured synthesis integrated the 15 included studies into six translational-readiness domains: external validation, generalizability, reproducibility and annotation heterogeneity, federated learning and privacy-preserving training, transparency and explainability, and clinical implementation readiness. This approach was chosen because the included studies were methodologically heterogeneous and could not be meaningfully reduced to a single quantitative endpoint. Instead of treating external performance as a uniform construct, the synthesis focused on the specific ways in which each study contributed to the broader question of whether dental AI models are moving toward responsible clinical use.

External validation was the foundational domain and was represented by all included studies [6,7,8,9,10,11,13,14,24,25,26,27,28,29,30]. This was a defining feature of the final corpus, because studies limited to internal train–validation–test performance had already been excluded. However, the form of external validation varied considerably. Some studies used independent patient cohorts or image repositories [6,10,11,24,25,30], whereas others relied on multicenter, multi-device, or cross-dataset testing [7,8,9,13,14,26,27,28,29]. As a result, the simple presence of an external test set did not imply the same level of translational maturity across studies.

Generalizability was the second most consistently represented domain. It was most explicitly addressed in studies that tested models across different centers, CBCT systems, radiographic acquisition technologies, clinical cohorts, or benchmark datasets [7,8,9,13,14,25,26,27,28,29,30]. These studies showed that performance could often be retained to a useful degree outside the development environment, but they also showed that transportability was conditional rather than automatic. Device differences, image-quality variation, labeling conventions, and external cohort characteristics frequently influenced performance, sometimes producing measurable attenuation relative to internal results [8,25,26,27].

Reproducibility and annotation heterogeneity were much less mature than external validation. The clearest evidence came from the AKUDENTAL study, which explicitly compared performance across multiple panoramic-radiograph datasets and demonstrated that annotation differences could materially affect apparent model performance [27]. Rubak et al. provided complementary evidence by showing that labeling inaccuracy, image noise, and faulty clients affected segmentation performance under distributed learning conditions [14]. Other studies contributed more limited reproducibility signals through code sharing, request-based data access, public repositories, or well-defined external datasets [10,11,26,29,30]. Overall, this domain remained a major weakness of the literature: many models were externally tested, but far fewer were independently reproducible.

Federated learning and privacy-preserving development were represented only by Schneider et al. and Rubak et al. [13,14]. Despite the small number of studies, this domain was important because it addressed a translational challenge that conventional external validation does not solve: how to build more generalizable models when raw imaging data cannot be freely pooled across institutions. Both studies showed that federated learning could outperform isolated local training and approach the performance of centralized learning, although sensitivity to center-level heterogeneity and data-quality problems remained evident [13,14].

Transparency and explainability were addressed inconsistently. Fang et al. used gradient-weighted class activation mapping to visualize model attention during radiographic quality assessment [8], while some other studies improved transparency through open or partially open resources, such as publicly available code, web-accessible models, or dataset repositories [10,11,24,26,27,30]. However, formal explainability methods were uncommon, confidence intervals were not always consistently reported, and many studies did not provide the code, trained weights, or full datasets needed for full external verification. In practice, transparency was often stronger in reporting the validation structure than in enabling genuine computational reproducibility.

Clinical implementation readiness was the least uniformly represented domain and was concentrated in a smaller subset of studies. The strongest examples were those that moved beyond technical validation alone, such as the external clinical validation of commercial decision-support systems for caries and periodontal bone loss [6], the multicenter PRG-Net study that evaluated dentist performance with and without AI assistance [7], the quality-control study that explicitly linked model outputs to radiographic workflow [8], the CBCT segmentation study that examined refinement burden under real-world conditions [9], and the intraoral-scan caries study that compared AI outputs with practitioner assessment [25]. Most other studies remained primarily technical, even when externally validated.

Taken together, the structured narrative synthesis showed that the current literature has moved beyond purely internal algorithmic performance, but translational readiness remains uneven across domains. External validation and generalizability are increasingly represented, whereas reproducibility, privacy-preserving scalability, explainability, and implementation readiness remain less consistently developed. In this sense, the strongest pattern across the included studies was not the presence of a single best-performing methodological strategy, but the emergence of a gradual transition from proof-of-concept modeling toward clinically transferable dental AI.

**Figure 3:**
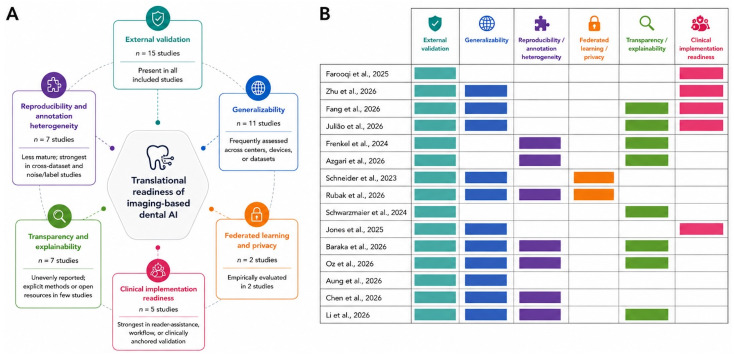
Structured synthesis of translational readiness in imaging-based dental artificial intelligence. (**A**) shows the six functional domains used to synthesize translational readiness across the included studies and the number of studies contributing to each domain. (**B**) maps each study to the domains it addressed empirically. Column colors indicate the six domains: teal, external validation; blue, generalizability; purple, reproducibility and annotation heterogeneity; orange, federated learning and privacy; green, transparency and explainability; and pink, clinical implementation readiness. Colored cells indicate a direct empirical contribution to the corresponding domain, whereas empty cells indicate that the domain was not directly addressed. Author–year labels in Panel B correspond to the following references: Farooqi et al., 2025 [6]; Zhu et al., 2026 [7]; Fang et al., 2026 [8]; Julião et al., 2026 [9]; Frenkel et al., 2024 [10]; Azgari et al., 2026 [11]; Schneider et al., 2023 [13]; Rubak et al., 2026 [14]; Schwarzmaier et al., 2024 [24]; Jones et al., 2025 [25]; Baraka et al., 2026 [26]; Oz et al., 2026 [27]; Aung et al., 2026 [28]; Chen et al., 2026 [29]; and Li et al., 2026 [30].

Figure 3 summarizes how the included studies contributed to each translational-readiness domain and illustrates the uneven development of external validation, generalizability, reproducibility, privacy-preserving learning, transparency, and clinical implementation readiness.

### 3.11. Exploratory Meta-Analysis/Feasibility of Quantitative Synthesis

Exploratory meta-analysis was considered for three candidate outcome families: external-validation AUC in detection or classification studies, external-validation segmentation performance using Dice similarity coefficient, and internal-to-external performance change. After reviewing the extracted data, quantitative pooling was not performed because the studies did not provide a sufficiently comparable set of tasks, units of analysis, reference standards, metrics, and uncertainty estimates.

For classification and detection studies, several articles reported external AUC or related discrimination metrics, but the clinical targets differed substantially. These included palatal radicular groove diagnosis and subtype classification in CBCT scans [7], technical-error classification and quality grading of periapical radiographs [8], root-number detection in maxillary premolars using panoramic radiographs with CBCT confirmation [11], and caries detection or classification in dental photographs [10,24]. Although these outcomes were all externally validated, they represented different diseases or technical tasks, different imaging modalities, and different reference standards. A pooled AUC would therefore have combined conceptually distinct diagnostic problems and would not have produced a clinically interpretable summary estimate.

A similar limitation was observed for segmentation outcomes. Dice similarity coefficient was reported or extractable in several segmentation studies, including posterior-tooth segmentation in CBCT scans [9], tooth segmentation in panoramic radiographs under federated or distributed learning settings [13,14], mixed-dentition CBCT segmentation of pulp and dental hard tissues [26], and multiview segmentation of teeth and gingiva in three-dimensional dental scans [28]. However, these studies segmented different anatomical structures, used different imaging modalities, evaluated different model families, and applied different validation frameworks. Even when the same metric was reported, the meaning of a Dice coefficient was not directly interchangeable across tooth segmentation, pulp segmentation, gingival segmentation, and federated panoramic-radiograph segmentation.

Internal-to-external performance change was also assessed for feasibility because it directly reflected the translational question of whether model performance was preserved outside the development environment. Several studies reported internal and external results or described external performance attenuation [8,10,11,24,25,26,27,30]. However, the available comparisons were expressed using different metrics, including AUC, accuracy, sensitivity, specificity, Dice similarity coefficient, Hausdorff distance, intersection over union, mean absolute error, root mean square error, and mean average precision. In addition, confidence intervals or standard errors were not consistently reported for both internal and external estimates. As a result, a pooled internal-to-external performance drop would have required assumptions that were not supported by the available data.

For these reasons, exploratory quantitative synthesis was judged to be inappropriate for the current dataset. The decision not to pool was not based on an insufficient number of included studies, but on the absence of methodologically compatible subgroups that shared the same task, imaging modality, unit of analysis, reference standard, and statistical metric. This heterogeneity also limited direct interpretation across studies, because the same numerical metric could have different clinical meanings depending on whether it was calculated for a diagnostic classification, anatomical segmentation, measurement task, image-level output, tooth-level output, or patient-level decision. The findings were therefore retained in the structured narrative synthesis, where the direction and nature of external performance, generalizability, reproducibility, and implementation readiness could be interpreted without forcing clinically heterogeneous outcomes into a single summary estimate. The feasibility assessment for each candidate quantitative synthesis is summarized in Table 7.

### 3.12. Certainty of Evidence

The certainty of evidence was summarized by functional domain using a GRADE-informed approach [20]. A single global certainty rating was not assigned because the included studies differed substantially in task, imaging modality, unit of analysis, validation structure, reference standard, and performance metric. Instead, certainty judgments were anchored to the specific translational question addressed by each domain: whether the available evidence supports external validation, generalizability, reproducibility, privacy-preserving learning, transparency, or clinical implementation readiness of imaging-based dental AI models.

Overall certainty ranged from very low to moderate across domains. The highest confidence was assigned to the presence of external validation, because all included studies evaluated performance beyond purely internal model development. However, confidence was reduced when external samples were small, based on independent image repositories with limited patient-level metadata, or not accompanied by complete uncertainty estimates. Certainty was lower for reproducibility, transparency, and clinical implementation readiness because open code, trained models, datasets, standardized reporting, and real-world workflow evaluation were inconsistently reported. The domain-level certainty assessment is summarized in Table 8.

## 4. Discussion

This systematic review showed that imaging-based dental AI has begun to move beyond internal model performance, but the evidence remains uneven across the translational pathway. All included studies incorporated some form of external validation, independent testing, multicenter assessment, cross-dataset evaluation, or federated-learning design. This represents a meaningful shift from the earlier dental AI literature, where model development frequently relied on internal train–test partitions, single-center datasets, or cross-validation without robust assessment in independent data [1,2]. However, the present findings also show that external validation alone does not establish implementation readiness. The strength of translational evidence depended on the level of dataset independence, the diversity of imaging devices and clinical settings, the stability of reference annotations, the availability of reproducibility resources, and the extent to which model outputs were evaluated in clinically meaningful workflows.

Previous reviews have mapped the broad expansion of machine learning in dentistry and have consistently highlighted heterogeneity in tasks, data sources, model architectures, validation strategies, and reporting quality [1,2]. Disease-specific evidence in caries detection has also suggested promising diagnostic performance but substantial variability across datasets, imaging modalities, annotation procedures, and reporting standards, with limited external validation in earlier studies [3]. More recently, systematic evidence addressing explainability, bias, and generalizability has emphasized that trustworthiness attributes are increasingly recognized in dental AI, although the evidence remains heterogeneous across domains and outcomes [4]. The present review differs from those earlier syntheses by restricting the evidence base to imaging-based dental AI studies that empirically tested at least one dimension of clinical transferability beyond internal development. Therefore, the central question was not whether dental AI can achieve high performance in controlled datasets, but whether these models have been evaluated under conditions that approximate clinical translation.

A key finding was that external validation was present across all included studies, but its meaning varied substantially. Some studies used independent patient cohorts or image datasets, whereas others evaluated models across centers, devices, acquisition protocols, public benchmark datasets, or federated-learning environments. These designs are not interchangeable. A model tested on an independent image repository may demonstrate a useful degree of external performance, but it does not face the same translational challenge as a model evaluated across multiple centers, scanner systems, annotation protocols, or distributed institutional datasets. This distinction is important because the clinical reliability of dental AI depends on its ability to remain stable when image acquisition, patient characteristics, disease spectrum, or annotation practices differ from those used during model development.

Accordingly, the synthesis assigned greater interpretive weight to multicenter, multi-device, cross-dataset, and federated/distributed validation designs than to single-cohort or independent image-repository validation.

Several studies provided stronger evidence of transportability by testing models across multicenter or multi-device conditions. For example, multicenter cone–beam computed tomography validation, periapical radiograph quality assessment across acquisition settings, multi-system cone–beam computed tomography segmentation, and distributed panoramic-radiograph segmentation directly addressed forms of domain shift that are likely to occur in real dental practice [7,8,9,13,14]. Cross-dataset studies further showed that performance can vary when datasets differ not only in image source but also in annotation structure and labeling conventions [27]. These findings support a central conclusion of this review: generalizability is not a binary property of a model but a context-dependent characteristic shaped by the validation environment.

At the same time, the evidence does not yet support the assumption that externally validated dental AI models are broadly transferable across all clinical settings. External samples were often smaller than development datasets, and some studies relied on image repositories or open-source datasets with limited patient-level or center-level information. In such cases, external testing remains useful, but it may not fully capture the variability of routine dental practice. This is particularly relevant for tasks such as caries detection, segmentation of anatomical structures, root morphology assessment, and gingival inflammation grading, where performance may depend on imaging quality, anatomical complexity, lesion spectrum, and operator-dependent acquisition factors.

The most persistent weakness across the included literature was reproducibility. Many studies reported external validation, but far fewer provided the resources needed for independent verification, such as open code, trained model weights, full datasets, data partitions, preprocessing scripts, or detailed annotation protocols. This distinction matters because a model can be externally validated within a published study and still remain difficult to reproduce, benchmark, or adapt in another clinical environment. In this review, reproducibility was strongest when studies provided public datasets, open code, web-accessible models, or repository-based access, but these features were not consistently available across the corpus.

Annotation heterogeneity emerged as a particularly important issue. Dental AI models often depend on labels produced by experts, consensus groups, manual segmentations, refined automatic segmentations, or clinical documentation. These reference standards are clinically reasonable, but they are not always equivalent across datasets or institutions. The AKUDENTAL study was especially informative because it showed that cross-dataset performance can be affected by differences in annotation definitions and labeling granularity [27]. Similarly, Rubak et al. [14] demonstrated that label inaccuracy, image noise, and faulty clients can alter segmentation performance under distributed learning conditions. These findings suggest that apparent model failure in external datasets may sometimes reflect inconsistencies in the target labels rather than only weaknesses of the algorithm.

The reporting limitations observed in this review are consistent with broader concerns in AI model evaluation. Transparent reporting frameworks such as TRIPOD + AI emphasize that machine-learning models require clear descriptions of data sources, predictors or inputs, outcomes, missing data, validation procedures, performance measures, and reproducibility elements to allow readers to judge model reliability and applicability [31]. Although TRIPOD + AI is focused on prediction models, its underlying principle is directly relevant to imaging-based dental AI: without transparent reporting of how data were selected, partitioned, annotated, processed, and externally tested, performance estimates remain difficult to interpret and reproduce.

Privacy-preserving learning was represented by only two included studies, both focused on tooth segmentation in panoramic radiographs [13,14]. Despite this small number, this domain is highly relevant to the translational future of dental AI. Dental radiographs and three-dimensional dental records may contain individualizing anatomical and restorative patterns, and the direct pooling of multi-institutional imaging data may be restricted by ethical, legal, regulatory, or institutional constraints. Federated learning addresses this challenge by enabling collaborative model training without direct exchange of raw patient images [12].

The included federated-learning studies suggest that distributed training can improve over isolated local learning and may approach centralized learning under some conditions [13,14]. However, they also show that privacy-preserving learning is not automatically equivalent to robust clinical generalization. Performance remained sensitive to client-level heterogeneity, label noise, image noise, and data-quality problems. Therefore, federated learning should be interpreted as an emerging translational strategy rather than a mature implementation standard. Its success will require more than technical aggregation of model updates; it will also require governance structures, annotation harmonization, quality-control procedures, monitoring of anomalous clients, and institutional capacity to support secure distributed model development.

Beyond technical validation, successful clinical implementation depends on several translational factors that were only partially addressed in the included literature. Regulatory approval pathways, post-market surveillance requirements, reimbursement mechanisms, integration with existing clinical information systems, workflow compatibility, clinician acceptance, professional liability, and patient trust all influence whether an AI model ultimately reaches routine practice. Even highly accurate and externally validated systems may face substantial barriers if they increase workflow burden, lack interoperability with imaging or electronic health record platforms, generate outputs that clinicians do not trust, or create uncertainty regarding responsibility for AI-assisted decisions. These considerations are particularly relevant in dentistry, where implementation frequently occurs in small private practices with variable digital infrastructure and limited technical support. Therefore, clinical implementation readiness should be viewed as a multidimensional construct that extends beyond external validation and includes regulatory, organizational, economic, and human-factor considerations.

The clinical implication of these findings is that externally validated dental AI should currently be viewed mainly as decision support rather than as autonomous clinical decision-making. The strongest near-term applications are likely to be tasks in which AI can assist clinicians by prioritizing images, identifying suspicious findings, supporting segmentation or measurement, improving quality control, or reducing repetitive workload. However, clinical adoption should remain cautious until models demonstrate reproducibility, workflow compatibility, safety, and usefulness in real clinical environments.

Clinical implementation readiness was less developed than external validation or generalizability. Moreover, very few studies reported information relevant to regulatory status, reimbursement considerations, workflow integration, user acceptance, liability frameworks, or patient-centered implementation outcomes. A small subset of studies moved beyond technical validation by evaluating commercial AI decision-support systems, clinician-AI comparisons, reader assistance, workflow-oriented quality control, segmentation refinement burden, or practitioner agreement [6,7,8,9,25]. These studies are important because they begin to address how AI outputs may interact with clinical judgment, diagnostic workflow, or task efficiency. However, most included studies remained primarily technical, even when externally validated.

The gap between external validation and clinical implementation is substantial. A model may perform well on an external dataset but still fail to improve clinical workflow, decision quality, patient outcomes, efficiency, or safety. Taken together, these findings suggest that the current role of dental AI is more consistent with decision support than autonomous clinical decision-making. Many of the included studies demonstrated the potential of AI to assist image interpretation, quality control, segmentation, or diagnostic assessment, but very few evaluated whether AI could safely replace clinician judgment in real-world settings. Therefore, the most realistic near-term implementation pathway is likely to involve human–AI collaboration, in which AI systems help clinicians manage large volumes of imaging data, identify relevant findings, and improve efficiency, while final clinical decisions remain the responsibility of trained professionals.

An additional challenge is that numerical model performance does not automatically translate into clinical utility. Metrics such as AUC, sensitivity, specificity, negative predictive value, Dice similarity coefficient, or Hausdorff distance should be interpreted in relation to the intended clinical task, the consequences of diagnostic or segmentation errors, and the context in which the model will be used. For example, a negative predictive value that may be useful for ruling out disease in a screening context does not necessarily imply suitability for treatment planning, while a high segmentation Dice similarity coefficient may still be insufficient if clinically relevant anatomical boundaries are inaccurately delineated. Because the included studies addressed highly heterogeneous clinical applications, universally applicable thresholds for clinical acceptability could not be defined. Instead, the findings of this review should be interpreted as evidence of technical and translational performance rather than definitive evidence of clinical utility. The DECIDE-AI reporting guideline is relevant here because it focuses on early-stage clinical evaluation of AI-based decision-support systems, including clinical setting, user interaction, human factors, implementation context, and safety considerations [32]. Very few dental AI studies in this review approached that level of implementation assessment. Similarly, when future studies aim to test AI systems as clinical interventions, reporting should align with CONSORT-AI to ensure adequate description of the AI intervention, human–AI interaction, trial context, error handling, and outcome assessment [33].

Therefore, the evidence synthesized here supports a cautious interpretation: within the selected subset of externally tested studies, imaging-based dental AI shows encouraging movement toward more rigorous validation, but this should not be interpreted as broad clinical maturity. The gap between external validation and implementation-ready AI remains substantial because reproducibility, workflow evaluation, regulatory considerations, clinician acceptance, and post-deployment monitoring were inconsistently addressed.

Exploratory quantitative synthesis was considered but not performed because the available studies did not provide methodologically compatible subgroups. This decision should not be interpreted as a weakness of the review; rather, it reflects the structure of the evidence. Pooling external area under the curve values across palatal radicular groove diagnosis, radiographic quality control, root-number detection, and caries classification would have produced a summary estimate without a coherent clinical meaning. Similarly, Dice similarity coefficients from cone–beam computed tomography tooth segmentation, panoramic-radiograph segmentation, mixed-dentition pulp and hard-tissue segmentation, and three-dimensional scan segmentation are not directly interchangeable, even when they share the same metric name.

The same issue applied to internal-to-external performance change. Several studies reported internal and external results, but they used different metrics, units of analysis, imaging modalities, and validation structures. Confidence intervals or standard errors were also not consistently available for paired comparisons. A pooled estimate would therefore have required assumptions that were not supported by the reported data. The structured narrative synthesis was better suited to this evidence base because it allowed the review to preserve clinically meaningful distinctions among validation type, modality, task, reference standard, reproducibility, and implementation relevance.

The findings of this review suggest three priority directions for future dental AI research. First, external validation should be strengthened prospectively and should involve patient-level, center-level, device-level, or cross-dataset independence whenever possible, because random image-level splitting or poorly described external datasets may overestimate transportability. Second, reproducibility and interpretability should be improved through uncertainty estimates, explicit leakage-prevention procedures, transparent annotation protocols, public code or model weights when feasible, and clear reporting of the unit of analysis and metric calculation. This is particularly important when multiple images, teeth, surfaces, or slices originate from the same patient, or when performance is calculated at pixel, tooth, image, or patient level. Third, studies claiming clinical relevance should move toward implementation-oriented designs, including reader studies, workflow analyses, prospective silent-mode evaluation, multicenter clinical validation, clinician–AI interaction studies, cost and resource assessments, and post-deployment monitoring. In this sense, the next stage of dental AI should be judged not only by whether models perform well, but by whether they remain reliable, reproducible, privacy-compatible, and useful across real-world clinical environments.

This review has several strengths. It focused on a clearly defined translational question and restricted the included corpus to imaging-based dental AI studies that went beyond internal model development. This allowed the synthesis to examine external validation, generalizability, reproducibility, privacy-preserving learning, transparency, and implementation readiness as distinct but related domains. The structured narrative synthesis provided a planned way to interpret methodologically heterogeneous studies without forcing incompatible outcomes into a single pooled estimate. The review also incorporated risk-of-bias, reporting-quality, and GRADE-informed certainty assessments aligned with the specific nature of dental AI validation studies.

Several limitations should also be considered. The included evidence was recent and relatively small, and the studies differed substantially in clinical task, imaging modality, unit of analysis, model architecture, reference standard, and performance metric. Because eligibility required at least one translational-validation dimension beyond internal model development, the included corpus represents a selected and relatively advanced subset of imaging-based dental AI research rather than the full dental AI literature. This design was appropriate for the review question, but it may overestimate the maturity of the broader field if interpreted as representing all dental AI studies, many of which remain limited to internal validation. If internally validated studies without external testing were considered in the denominator, the overall translational readiness of dental AI would likely be lower than suggested by the included evidence. Some judgments on transparency, leakage prevention, and reproducibility depended on what authors reported, and independent verification was not always possible. The review excluded non-imaging dental AI studies from the final corpus to preserve methodological coherence, even though some non-imaging models may provide useful lessons about external validation and transportability. Finally, because quantitative pooling was not appropriate, the conclusions rely on structured narrative synthesis rather than pooled performance estimates.

Overall, the findings support a cautious interpretation of the current evidence and show that translational readiness in dental AI depends on more than external validation alone.

## 5. Conclusions

Within the selected subset of imaging-based dental AI studies that had already attempted external testing or related translational validation, this systematic review showed movement beyond internal algorithmic performance toward external validation, multicenter and multi-device testing, cross-dataset evaluation, and privacy-preserving learning. However, the field remains in an early translational phase rather than being fully implementation ready. The strength of evidence differs substantially according to the level of external validation, with multicenter, multi-device, cross-dataset, and federated/distributed designs providing stronger evidence of transportability than single-cohort or image-repository validation.

Externally validated dental AI models should not be considered implementation-ready solely on the basis of favorable performance metrics. Future studies should demonstrate that models remain reliable, reproducible, privacy-compatible, clinically useful, and workflow-compatible across diverse real-world settings. Progress toward clinical adoption will require transparent reporting, robust external validation, regulatory and governance planning, clinician acceptance, patient trust, and post-development evaluation.

## Figures and Tables

**Table 1:** General characteristics of the included studies.

Authors, Year, Country	Dental Domain	Imaging Modality/Data Source	AI Task	Sample Size	Unit of Analysis	Model Type	Reference Standard	Validation Type
Zhu et al., 2026, China [7]	Endodontics/palatal radicular grooves	CBCT scans	Tooth segmentation, PRG diagnosis, and PRG subtype classification	Internal PRG dataset: 229 PRG teeth; three external datasets: 81 PRG teeth; clinical validation: 30 CBCT scans/884 teeth	Tooth/CBCT scan	PRG-Net deep-learning framework	Expert-established PRG diagnosis and subtype classification	Multicenter validation with one internal site and three external centers
Fang et al., 2026, China [8]	Dentomaxillofacial radiology/radiographic quality control	Periapical radiographs	Technical-error classification and radiographic quality grading	3510 periapical radiographs from two centers	Radiograph	Multi-task deep-learning model with EfficientNet backbone	Expert quality grading using NRPB and CGDent standards and technical-error annotations	Internal and external validation across centers and acquisition systems
Julião et al., 2026, Brazil [9]	Oral radiology/CBCT segmentation	CBCT scans acquired with five CBCT systems	Automated segmentation of posterior teeth	190 CBCT scans from 190 patients; 1005 posterior teeth	Tooth/CBCT scan	AI-based 3D segmentation software	Refined automatic segmentation and manual segmentation in a subset	External validation across multiple CBCT systems and clinical centers
Azgari et al., 2026, Turkey [11]	Endodontics/root morphology	Panoramic radiographs with CBCT reference	Detection of root number in maxillary premolars	925 premolars from 350 patients; independent external validation set: 148 premolars	Tooth	AlexNet, DenseNet-121, EfficientNet-B0, and ensemble CNN model	CBCT-based root-number assessment	Independent patient-level external validation and comparison with an experienced endodontist
Rubak et al., 2026, Denmark/Finland [14]	Dental radiography/tooth segmentation	Panoramic radiographs from six institutions	Tooth segmentation under heterogeneous data-quality conditions	2066 panoramic radiographs	Radiograph/tooth segmentation mask	Attention U-Net trained using federated, centralized, and local learning	Tooth segmentation annotations	Federated-learning evaluation with centralized and local learning comparators; robustness testing for label noise, image noise, and faulty clients
Baraka et al., 2026, Egypt [26]	Pediatric dentistry/mixed dentition segmentation	CBCT scans	Segmentation of pulp, primary dental hard tissues, and permanent dental hard tissues	151 CBCT scans: 105 internal scans and 46 external scans	CBCT scan/tooth structure/pulp structure	CNN-, Transformer-, and Mamba-based 3D segmentation models, including ResEncM, U-Mamba Bot/Enc, WNet, UNETR, and SegResNet	Expert-annotated segmentation	Independent external CBCT testing across heterogeneous acquisition protocols
Oz et al., 2026, Turkey [27]	Dental radiography/instance segmentation	Panoramic radiographs	Tooth and restoration instance segmentation, object detection, and semantic segmentation	AKUDENTAL dataset: 333 panoramic images with 9956 annotated structures; external cross-dataset testing on Tufts, DENTEX, and Dual-labeled datasets	Image/tooth instance/restoration instance	UNet, DeepLabV3+, YOLOv11, and Mask R-CNN	Expert-supervised annotations	Cross-dataset generalizability and annotation-heterogeneity analysis
Aung et al., 2026, Thailand [28]	Orthodontics/digital model analysis	3D intraoral scans	Multiview segmentation of teeth and gingiva	1200 public dental models and 29 clinical intraoral scans	Intraoral scan/tooth/gingival segment	Multiview Mask2Former with Swin-B Transformer backbone	Manual segmentation by two experienced clinicians	Clinical generalization testing on real-world intraoral scans
Chen et al., 2026, China [29]	Periodontology/gingival inflammation	Standard oral RGB images	Gingival inflammation grading	2300 internal gingival-inflammation images; 1096 open-source external images	Image/gingival region	HFSA-DETR model based on dual-domain spatial-frequency analysis and adaptive sparse attention	Gingival-inflammation grade labels	External validation using an open-source dataset under diverse imaging conditions
Li et al., 2026, China [30]	Orthodontics/dental morphology measurement	Standardized 2D occlusal photographs with 3D intraoral scan reference	Estimation of single-tooth mesiodistal width	14,403 teeth: 12,079 internal teeth and 2328 teeth in an independent external test set	Tooth	Two-stage deep-learning framework with keypoint detection and depth-informed regression	Mesiodistal crown widths derived from 3D intraoral scans	Independent external dataset validation within a standardized photographic workflow
Farooqi et al., 2025, United States [6]	Caries and periodontal bone loss	Full-mouth radiographs and longitudinal clinical records	External validation of AI-based clinical decision-support systems	90 patients with full-mouth radiographs and 6–12 months of follow-up	Patient/radiographic finding	Two FDA-cleared commercial AI decision-support systems	Longitudinal clinical documentation with calibrated examiner adjudication	External clinical validation using retrospective longitudinal ground truth
Jones et al., 2025, Australia [25]	Pediatric dentistry/caries detection	Intraoral scans from children	Caries detection and segmentation	Development cohort: 332 carious teeth; external cohort: 119 carious teeth	Tooth/lesion/3D scan-derived image representation	Attention U-Net	Dental practitioner assessment using ICCMS-based annotation	Independent external cohort validation and diagnostic agreement with practitioners
Frenkel et al., 2024, Germany [10]	Dental caries	Dental photographs	Caries detection, classification, localization, and segmentation	718 independent dental photographs: 535 carious and 183 noncarious teeth	Photograph/tooth/lesion class/lesion area	Freely accessible AI-based caries model	Dental workgroup consensus classification	External validation using an independent image dataset
Schwarzmaier et al., 2024, Germany [24]	Early childhood caries	Dental photographs of anterior deciduous teeth	ECC detection and carious-lesion classification	143 anonymized photographs: 107 ECC and 36 controls	Photograph/anterior deciduous tooth	Freely accessible AI-based caries model	Visual evaluation by dental study group	External validation using independent dental photographs
Schneider et al., 2023, Germany/international multicenter dataset [13]	Dental radiography/tooth segmentation	Panoramic radiographs from nine international centers	Tooth segmentation	4177 panoramic radiographs from nine centers	Radiograph/tooth segmentation mask	Deep-learning tooth-segmentation model trained under federated, local, and centralized learning paradigms	Tooth segmentation by one expert and verification by a second independent expert	Federated, centralized, and local learning comparison; generalizability assessed on combined multicenter test data

AI, artificial intelligence; CBCT, cone–beam computed tomography; CGDent, College of General Dentistry; CNN, convolutional neural network; ECC, early childhood caries; FDA, Food and Drug Administration; HFSA-DETR, Hybrid-Frequency Self-Attention Detection Transformer; ICCMS, International Caries Classification and Management System; NRPB, National Radiological Protection Board; PRG, palatal radicular groove; R-CNN, region-based convolutional neural network; RGB, red–green–blue; U-Net, U-shaped convolutional neural network; YOLO, You Only Look Once; 2D, two-dimensional; 3D, three-dimensional.

**Table 2:** External validation characteristics of the included studies.

Study	External Dataset/Cohort	Level of Independence	Validation Setting	Main External Metric(s)	Comparator/Reference	Internal-to-External Change Reported?
Zhu et al., [7]	Three external CBCT datasets from independent centers, plus an independent clinical validation set of 30 CBCT scans	Center-level and clinical-validation independence	Multicenter CBCT validation of PRG segmentation, diagnosis, and subtype classification	AUC for PRG diagnosis and subtype classification; diagnostic/classification accuracy; dentist performance with and without PRG-Net assistance	Expert-established PRG diagnosis and classification; visual diagnosis by junior and senior dentists	Yes; internal and external AUCs were reported, and clinical performance was compared with and without AI assistance
Fang et al., [8]	External periapical radiographs from another center and acquisition system	Center- and device-level independence	External testing across PSP/CMOS-related acquisition differences	External AUC, sensitivity, specificity, precision, accuracy, and F1-score; technical-error AUC 0.842–0.978; quality-grading AUC 0.940–0.960	Expert quality grading using NRPB and CGDent standards; technical-error annotations	Yes; internal and external test-set performance were reported
Julião et al., [9]	CBCT scans from 190 patients acquired using five CBCT systems	Patient-, device-, and center-level independence	Generalizability testing of AI-based posterior-tooth segmentation software	IoU 0.93–0.96; DSC 0.96–0.98; precision 0.99–1.00; recall 0.94–0.96; accuracy 0.98–0.99; MAD 0.05–0.07; RMSE 0.06–0.14	Refined automatic segmentation and manual segmentation in a subset	Not as a conventional internal-to-external drop; the study primarily evaluated generalizability across devices and clinical conditions
Azgari et al., [11]	Independent external validation set of 148 maxillary premolars from new patients	Patient-level external validation	External testing of root-number classification on panoramic radiographs using CBCT as reference	Accuracy, sensitivity, specificity, F1-score, and AUC; ensemble external accuracy 0.87	CBCT-based root-number assessment; experienced endodontist comparator	Yes; cross-validation and independent external validation results were reported
Rubak et al., [14]	Panoramic radiographs from six institutions under distributed learning scenarios	Institution-level distributed validation	Federated, centralized, and local learning under label-noise, image-noise, and faulty-client scenarios	Segmentation performance metrics under FL, CL, and LL; robustness to data-quality perturbations	Tooth segmentation annotations; CL and LL comparators	Yes; learning paradigms and robustness scenarios were compared
Baraka et al., [26]	External set of 46 CBCT scans	Dataset-level external validation	External CBCT testing for mixed-dentition segmentation	External DSC and HD95; ResEncM achieved DSC 0.8464 ± 0.1226 and HD95 4.9078 ± 4.9058	Expert-annotated segmentation	Yes; external testing showed lower performance than internal testing
Oz et al., [27]	Tufts, DENTEX, and Dual-labeled panoramic-radiograph datasets	Cross-dataset independence	Cross-dataset evaluation of tooth and restoration segmentation/detection	mAP and mAP@50; multiclass cross-dataset mAP ranged from 0.34 on DENTEX to 0.71 on the Dual-labeled dataset	External dataset annotations with different labeling protocols	Yes; cross-dataset performance differences were explicitly analyzed
Aung et al., [28]	MUOrtho clinical intraoral-scan dataset of 29 scans, in addition to TeethChallenge testing data	Dataset- and clinical-sample independence	Clinical generalization testing of multiview segmentation on real-world intraoral scans	Per-class DSC and mIoU; MUOrtho DSC values ranged from 0.757 to 0.967 across classes for the proposed model	Manual segmentation by two experienced clinicians; geometric and deep-learning baselines	Yes; performance was compared across public and clinical datasets
Chen et al., [29]	Open-source external dataset of 1096 gingival-inflammation images	Dataset-level external validation	Generalization testing for gingival-inflammation grading from oral RGB images	Precision, recall, mAP50, and mAP50:95; HFSA-DETR reported precision 95.2%, recall 93.6%, mAP50 95.8%, and mAP50:95 83.5% in model testing	Gingival-inflammation grade labels; comparison with Faster R-CNN, YOLO variants, and RT-DETR	Partly; the model was developed on an internal dataset and evaluated for generalization, but the internal-to-external change was not the central reported contrast
Li et al., [30]	Independent external test set of 2328 teeth	Dataset-level and tooth-level external validation	External validation of tooth-width estimation from standardized occlusal photographs	MAE 0.33 mm; RMSE 0.42 mm; high agreement with 3D scan-derived measurements	Mesiodistal crown widths from 3D intraoral scans	Yes; internal and external performance were reported and showed consistent results
Farooqi et al., [6]	Independent retrospective clinical cohort of 90 patients with full-mouth radiographs and 6–12 months of follow-up	Patient-level external validation	External clinical validation of two commercial AI decision-support systems	Concordance, specificity, and NPV; caries NPV 96.92% and 97.36%; periodontal bone loss NPV 80.64% and 75.20% for vendors A and B	Longitudinal clinical documentation with calibrated examiner adjudication	No; the study was designed as external validation rather than internal-to-external comparison
Jones et al., [25]	Second independent cohort of 119 carious teeth	Cohort-level external validation	External validation of caries detection using intraoral scans from children	IoU, sensitivity, specificity, precision, and diagnostic agreement; performance slightly declined on the external dataset	ICCMS-based annotations and dental-practitioner assessment	Yes; internal test and external cohort performance were compared
Frenkel et al., [10]	Independent dataset of 718 dental photographs retrieved from the internet	Independent image-dataset validation	External validation of a freely accessible caries model	Accuracy 92.0% for caries detection; classification accuracy 85.5–95.6%; sensitivity 42.9–93.3%; specificity 82.1–99.4%; AUC 0.702–0.909; localization accuracy 97.0%	Dental-team consensus reference standard	Yes; results were compared with previously published internal validation data
Schwarzmaier et al., [24]	Independent set of 143 anonymized photographs of anterior deciduous teeth	Independent image-dataset validation	External validation of a freely accessible ECC detection/classification model	ECC detection accuracy 97.2%; lesion-class accuracies 88.9–98.1%; sensitivities 68.8–98.5%; specificities 86.1–99.4%	Visual evaluation by the dental study group	Yes; external results were compared with the model’s previously published internal validation
Schneider et al., [13]	Panoramic radiographs from nine international centers	Multicenter distributed-data independence	Comparison of federated, local, and centralized learning for tooth segmentation	Tooth-wise F1-score and segmentation-related metrics; FL outperformed LL in most centers, while CL generally remained superior	Expert tooth segmentation verified by a second independent expert	Yes; local, federated, centralized, and combined multicenter test performance were compared

AI, artificial intelligence; AUC, area under the curve; CBCT, cone–beam computed tomography; CGDent, College of General Dentistry; CL, centralized learning; CMOS, complementary metal–oxide–semiconductor; DSC, Dice similarity coefficient; ECC, early childhood caries; FL, federated learning; HD95, 95th percentile Hausdorff distance; HFSA-DETR, Hybrid-Frequency Self-Attention Detection Transformer; ICCMS, International Caries Classification and Management System; IoU, intersection over union; LL, local learning; MAE, mean absolute error; MAD, mean absolute distance; mAP, mean average precision; mIoU, mean intersection over union; NPV, negative predictive value; NRPB, National Radiological Protection Board; PRG, palatal radicular groove; PSP, photostimulable phosphor; RGB, red–green–blue; RMSE, root mean square error; RT-DETR, Real-Time Detection Transformer.

**Table 3:** Generalizability features across centers, devices, and datasets.

Study	Number of Centers	Number/Type of Devices	Domain Shift Evaluated	Generalizability Context	Main Finding on Generalizability	Key Limitation
Farooqi et al. [6]	Not primarily center-based	Full-mouth radiographs; device details not central to the analysis	Commercial-system transportability to an independent clinical cohort	Clinical follow-up setting rather than device-shift testing	The systems showed high negative predictive value for ruling out caries and periodontal bone loss, supporting use as adjunctive screening tools under clinician supervision	Relatively small clinical cohort; device- and center-level domain shift were not the main focus
Zhu et al. [7]	Four centers: one internal and three external centers	CBCT scans; center-specific acquisition conditions	Multicenter domain shift for PRG segmentation, diagnosis, and classification	External centers tested whether PRG-Net remained useful outside the development site	PRG-Net maintained clinically useful external diagnostic and classification performance and improved dentists’ diagnostic consistency and efficiency	External datasets were smaller than the internal development dataset, and the task was restricted to a specialized endodontic condition
Fang et al. [8]	Two centers	Periapical radiographs acquired with different technologies, including PSP and CMOS-related acquisition differences	Center-level and device/acquisition shift	External testing involved differences in acquisition technology and radiographic workflow	The model maintained strong external performance for quality grading and several technical-error categories, but some attenuation occurred externally	External performance varied by technical-error type, suggesting sensitivity to image acquisition and anatomical complexity
Julião et al. [9]	Multiple clinical sources; analysis centered on five CBCT systems	Five CBCT systems	Multi-device CBCT generalizability	Different CBCT systems tested whether segmentation performance was stable across acquisition hardware	The AI-based segmentation software showed high overlap metrics across CBCT systems, although some teeth required refinement	Generalizability was evaluated for posterior teeth and for a specific segmentation software workflow
Frenkel et al. [10]	Not center-based	Dental photographs from an independent image repository	Independent image-dataset shift	Independent photographic images tested model transfer outside the original development dataset	The freely accessible caries model showed high performance for several detection and classification tasks	Limited patient-level metadata restricted assessment of population- or center-level transportability
Azgari et al. [11]	Not multicenter; patient-level external set	Panoramic radiographs with CBCT reference	Patient-level external validation	New patients tested whether root-number classification generalized beyond the development sample	The ensemble model showed good external performance and was compared with an experienced endodontist	The external set was patient-independent but not clearly multicenter or multi-device
Schneider et al. [13]	Nine international centers	Panoramic radiographs from multiple centers	Multicenter and distributed-data shift	Distributed panoramic-radiograph data tested whether federated learning improved cross-center segmentation	Federated learning improved over local learning in most centers and approached centralized learning	Centralized learning generally remained stronger, and center-level heterogeneity still influenced performance
Rubak et al. [14]	Six institutions	Panoramic radiographs from multiple institutions	Institution-level heterogeneity, image noise, labeling inaccuracy, and faulty-client effects	Robustness scenarios tested generalizability under imperfect multicenter data conditions	Federated learning was informative for distributed generalization, but performance was sensitive to labeling errors, image noise, and defective clients	Experimental perturbations may not fully reproduce all sources of real-world clinical heterogeneity
Schwarzmaier et al. [24]	Not center-based	Dental photographs of anterior deciduous teeth	Independent image-dataset shift	External dental photographs tested transferability of an ECC detection/classification model	The model showed high external performance for ECC detection and several lesion classes	External dataset was relatively small and anatomically focused on anterior deciduous teeth
Jones et al. [25]	Independent cohort design	Intraoral scans from children	Cohort-level external shift	External pediatric intraoral-scan data tested whether caries detection transferred across cohorts	External performance decreased compared with internal testing but remained clinically informative	External cohort was smaller than the development cohort, and performance may depend on scan quality and lesion representation
Baraka et al. [26]	Internal and external datasets; center count not the main reporting unit	CBCT scans with heterogeneous acquisition protocols	External CBCT dataset and acquisition-protocol shift	Heterogeneous CBCT acquisition tested segmentation robustness in mixed dentition	External performance was lower than internal performance, indicating that acquisition heterogeneity affects CBCT segmentation transportability	External validation included fewer scans than the internal dataset and focused on mixed dentition
Oz et al. [27]	Cross-dataset rather than center-based	Panoramic-radiograph datasets with different annotation protocols	Cross-dataset and annotation-domain shift	External benchmark datasets tested transfer across labeling conventions and dataset structures	Cross-dataset performance varied substantially, showing that annotation heterogeneity can strongly affect apparent generalizability	Differences in label definitions and annotation granularity limited direct comparability across datasets
Aung et al. [28]	Public dataset plus one clinical validation source	3D dental models and real-world intraoral scans	Public-to-clinical dataset shift	Clinical intraoral scans tested whether a model trained with public data transferred to real-world scans	The multiview segmentation model generalized to clinical intraoral scans, although performance differed across tooth and gingival classes	Clinical validation used a limited number of real-world scans
Chen et al. [29]	Internal dataset plus external open-source dataset	Standard oral RGB images	Dataset-level image-domain shift	External open-source RGB images tested generalization under different imaging conditions	The model achieved strong external grading performance and outperformed several comparator detection models	External validation depended on open-source image labels and may not fully capture patient-level or center-level independence
Li et al. [30]	Internal and independent external dataset	Standardized 2D occlusal photographs with 3D intraoral scan reference	Dataset-level shift within a standardized photographic workflow	External tooth-level testing assessed transfer within controlled occlusal-photograph acquisition	External measurement error remained low, suggesting transportability within standardized photographic conditions	Generalizability may depend on maintaining standardized image acquisition and measurement protocols

AI, artificial intelligence; CBCT, cone–beam computed tomography; CMOS, complementary metal–oxide–semiconductor; ECC, early childhood caries; PRG, palatal radicular groove; PSP, photostimulable phosphor; RGB, red–green–blue.

**Table 4:** Reproducibility, annotation heterogeneity, and data transparency.

Study	Cross-Dataset Evaluation	Annotation Heterogeneity Assessed	Code Availability	Dataset Availability	Model Availability	Main Reproducibility Issue
Zhu et al. [7]	Yes; multicenter external CBCT validation across three external centers	Not formally assessed as annotation heterogeneity; expert-established PRG diagnosis and subtype classification were used	Not publicly reported	Not publicly reported	Not publicly reported	Multicenter validation was strong, but independent reproduction is limited by lack of open data, code, and model resources
Fang et al. [8]	Yes; external validation across centers and acquisition systems	Not formally assessed; labels followed radiographic quality and technical-error standards	Not publicly reported	Not publicly reported	Not publicly reported	Reproducibility depends on access to private periapical-radiograph datasets and consistent application of quality-grading standards
Julião et al. [9]	Yes; generalizability across multiple CBCT systems	Partly; automatic segmentations were refined and a subset was manually segmented, but cross-dataset annotation heterogeneity was not the primary analysis	Not publicly reported	Not publicly reported	Evaluated AI software; open model access not reported	Reproduction is limited by dependence on the evaluated segmentation software and non-public CBCT datasets
Azgari et al. [11]	No formal cross-dataset comparison; independent patient-level external validation was performed	Not formally assessed	Publicly available source code for preprocessing, model training, and evaluation	Not public because of ethical and legal restrictions; available from the corresponding author on reasonable request	Trained model availability not clearly reported	Code availability supports reproducibility, but patient imaging data are not public
Rubak et al. [14]	Yes; distributed multicenter evaluation across six institutions	Yes; label manipulation and image-noise scenarios were experimentally evaluated	Not publicly reported	Not publicly reported	Not publicly reported	The study directly tested robustness to label inaccuracy and image noise, but external reproduction is limited by lack of open datasets and code
Baraka et al. [26]	Yes; internal and external CBCT testing	Not formally assessed across annotators or datasets	Available in a private GitHub repository and Alexandria University repository upon request	ToothFairy dataset public; study data available through private/institutional repositories upon request	Trained model availability not clearly reported	Partial resource availability supports verification, but broader reproducibility depends on request-based access and moderate external sample size
Oz et al. [27]	Yes; AKUDENTAL was evaluated against Tufts, DENTEX, and Dual-labeled datasets	Yes; annotation-protocol differences were a central finding	Publicly available through GitHub for non-commercial academic use	Publicly available through GitHub for non-commercial academic use	Baseline code and evaluation resources available; trained weights not clearly specified	Strongest reproducibility profile, but single-expert annotation and cross-dataset label inconsistency remain important limitations
Aung et al. [28]	Yes; public challenge data to clinical intraoral-scan testing	Not formally assessed; manual segmentation by experienced clinicians was used	Not publicly reported	Public challenge data used; clinical validation scans not reported as public	Not publicly reported	Clinical generalization was tested, but reproducibility is limited by small clinical validation set and lack of open clinical data/code
Chen et al. [29]	Yes; external open-source gingival-inflammation image dataset	Partly; internal annotations were performed by three specialists with high agreement, but cross-dataset label harmonization was not the main focus	Not publicly reported	Internal dataset not public; external validation dataset was open-source	Not publicly reported	Strong annotation control was reported internally, but reproducibility is limited by unavailable internal data and possible differences in external labels
Li et al. [30]	Yes; independent external tooth-level dataset within standardized workflow	Not formally assessed as annotation heterogeneity; measurements were referenced to 3D intraoral scans	Not clearly reported as public	Datasets reported as available in the TDE repository	Not publicly reported	Dataset availability improves transparency, but generalizability was tested within standardized photography rather than across heterogeneous devices
Farooqi et al. [6]	No cross-dataset comparison; external clinical validation cohort was used	Not formally assessed; adjudicated longitudinal clinical documentation was used	Not applicable/not publicly reported	Not publicly reported	Commercial AI systems evaluated; proprietary models not open	Reproducibility is limited by proprietary systems and non-public clinical follow-up data
Jones et al. [25]	Yes; independent external cohort of intraoral scans	Not formally assessed as annotation heterogeneity; ICCMS-based annotation was used	Not publicly reported	Not publicly reported	Not publicly reported	The external cohort strengthens validation, but reproducibility is limited by unavailable scan data, annotations, and model code
Frenkel et al. [10]	Yes; independent image-dataset external validation	Partly; dental-team consensus reference standard was used, but no formal cross-dataset label-harmonization analysis was performed	Not publicly reported	Data available upon reasonable request	AI model available as a web application	The model can be tested through the web application, but code and training data are not open
Schwarzmaier et al. [24]	Yes; independent image-dataset external validation	Partly; visual evaluation by a dental study group was used, but no formal annotation-heterogeneity analysis was performed	Not publicly reported	Not publicly reported	Freely accessible AI-based model evaluated through web application	External validation was feasible because the model was accessible, but reproducibility remains limited by lack of open code and dataset
Schneider et al. [13]	Yes; federated, local, and centralized learning across nine international centers	Not formally assessed as annotation heterogeneity; tooth segmentation was verified by a second expert	Not publicly reported	Not publicly reported	Not publicly reported	Multicenter federated design supports transportability, but privacy constraints limit open data release and independent reproduction

AI, artificial intelligence; CBCT, cone–beam computed tomography; DENTEX, dental enumeration and diagnosis benchmark dataset; ICCMS, International Caries Classification and Management System; PRG, palatal radicular groove; TDE, Tooth Distance Estimation.

**Table 5:** Explainability and reporting transparency of included studies.

Study	Explainability Method	Uncertainty Reporting	External Validation Clearly Described	Data Leakage Prevention Described	Main Transparency Limitation	CLAIM-Derived Reporting Profile
Farooqi et al. [6]	Not reported; the study evaluated outputs of commercial AI systems	Partly reported; performance estimates and diagnostic agreement were presented, but uncertainty was not comprehensive for all outputs	Yes; the independent retrospective clinical cohort with longitudinal follow-up was clearly described	Partly; the external cohort design reduced concern about development-test overlap, but proprietary model-development details were unavailable	Limited transparency of model architecture, training data, and decision logic because proprietary commercial systems were evaluated	Partial: clinical setting, index tests, reference standard, external validation, and performance metrics were described; model-level transparency was limited
Zhu et al. [7]	Visual diagnostic framework; no formal post-hoc explainability method clearly reported	Partly reported; performance was presented across internal and external datasets, but uncertainty was not uniform for all tasks	Yes; one internal site and three external centers were explicitly described	Partly; separation between internal and external centers was clear, but leakage-prevention procedures were not fully reproducible from the report	Limited open reproducibility of model, data, and implementation details	Partial to high: multicenter design, reference standard, task definition, validation structure, and clinical reader comparison were well described
Fang et al. [8]	Grad-CAM visualizations were used to support interpretation of model attention	Partly reported; multiple performance metrics were presented, but confidence intervals were not consistently available for all metrics	Yes; external validation across centers and acquisition systems was described	Partly; training and external testing were separated by center/acquisition source	Open reproducibility remained limited despite inclusion of visual explainability	High for study design, data source, task definition, validation, reference standards, and interpretability; limited by incomplete reproducibility resources
Julião et al. [9]	No formal explainability method reported; clinical assessment focused on segmentation refinement	Partly reported; segmentation metrics were presented, but uncertainty reporting was not uniform across all outputs	Yes; generalizability across multiple CBCT systems was the main objective	Partly; patient-level data structure and device-level testing were described, but leakage-prevention details were not extensive	Dependence on evaluated AI software and non-open CBCT datasets limited independent verification	Partial to high: imaging source, AI software, segmentation metrics, validation setting, and refinement analysis were described
Frenkel et al. [10]	No formal explainability method reported	Yes/partly; diagnostic metrics and uncertainty estimates were reported for several outcomes	Yes; an independent external photographic dataset was described	Partly; external image independence was clear, but patient-level independence could not be fully verified	Patient-level metadata and full computational reproducibility were limited	Partial: external dataset, model access, reference standard, and performance metrics were reported
Azgari et al. [11]	No formal explainability method reported	Partly reported; external diagnostic metrics were presented, but not all uncertainty estimates were consistently available	Yes; the independent patient-level external validation set was described	Yes/partly; separation of training/validation data and external patient-level testing was described	Imaging data access was restricted, limiting full independent replication despite stronger code transparency	High for validation structure, external testing, reference standard, and methodological transparency
Schneider et al. [13]	No formal explainability method reported; focus was on federated, local, and centralized segmentation performance	Partly reported; segmentation metrics were presented across learning paradigms and centers	Yes; the nine-center dataset and federated/local/centralized comparison were described	Partly; center-wise data separation and distributed learning design reduced direct data pooling and leakage risk	Privacy-preserving multicenter design limited open verification of underlying institutional data	Partial to high: multicenter data source, validation design, learning paradigms, and reference annotation were described
Rubak et al. [14]	No formal explainability method reported; robustness monitoring through client behavior was evaluated	Partly reported; performance across corruption scenarios was presented, but uncertainty reporting was not uniform for all comparisons	Yes; the six-institution federated, centralized, and local learning design was described	Partly; institution-level learning paradigms and robustness scenarios were specified	Strong robustness design, but limited transparency for independent external reproduction	High for robustness testing, data-quality perturbation design, and validation structure
Schwarzmaier et al. [24]	No formal explainability method reported	Partly reported; diagnostic performance was presented for ECC detection and lesion classes	Yes; an independent external dental-photograph dataset was described	Partly; external validation of a previously developed model reduced concern about development-test overlap	Limited transparency of dataset, code, and original model-development process	Partial: accessible model, external dataset, reference assessment, and performance metrics were described
Jones et al. [25]	No formal explainability method reported	Partly reported; internal and external performance metrics were presented	Yes; the external cohort of carious teeth from children was described	Partly; development and external cohorts were separated	Limited availability of scan data, annotations, and implementation details reduced reproducibility	Partial: external cohort, task, reference standard, and performance metrics were described
Baraka et al. [26]	No formal explainability method reported	Yes/partly; segmentation metrics and comparative model results were presented	Yes; internal and external CBCT datasets were described	Partly; internal and external CBCT testing were clearly distinguished	Transparency was improved by repository-based access, but reproducibility still depended on request-based resources	High for reporting guideline adherence, model comparison, segmentation metrics, external testing, and data-source description
Oz et al. [27]	No formal explainability method reported	Partly reported; cross-dataset performance metrics were presented	Yes; cross-dataset evaluation against Tufts, DENTEX, and Dual-labeled datasets was described	Partly; dataset partitioning and cross-dataset testing were described	The main transparency limitation was not reporting explainability, although dataset and benchmarking transparency were strong	High: annotation protocol, public benchmarking resources, external datasets, and cross-dataset reproducibility were clearly reported
Aung et al. [28]	No formal explainability method reported	Partly reported; segmentation metrics were presented across public and clinical datasets	Yes; clinical validation on real-world intraoral scans was described	Partly; public-to-clinical testing was described, but leakage-prevention details were not extensive	Small clinical validation set and incomplete implementation transparency limited reproducibility	Partial: task, model, external clinical testing, and reference segmentation were described
Chen et al. [29]	Attention-based architecture was used; no formal post-hoc explainability method clearly reported	Partly reported; detection/grading metrics were presented, but uncertainty estimates were not consistently provided	Yes; the external open-source image dataset was described	Partly; internal and external datasets were separated	Internal resource transparency and external label comparability remained limited	Partial to high: external testing, comparator models, annotation agreement, and performance metrics were reported
Li et al. [30]	No formal explainability method reported	Partly reported; measurement error and agreement metrics were presented	Yes; the independent external tooth-level dataset was described	Partly; internal and external test sets were separated within a standardized workflow	Code and model transparency were limited despite dataset-level availability	Partial to high: external validation, reference measurements, task-specific metrics, and dataset availability were reported

AI, artificial intelligence; CBCT, cone–beam computed tomography; CLAIM, Checklist for Artificial Intelligence in Medical Imaging; DENTEX, dental enumeration and diagnosis benchmark dataset; ECC, early childhood caries; Grad-CAM, gradient-weighted class activation mapping.

**Table 6:** Risk-of-bias and methodological-quality assessment of the included studies.

Study	Appraisal Approach	External Data Selection	Reference Standard/Annotation Quality	Validation and Leakage-Prevention Reporting	Reproducibility and Transparency	Overall Appraisal Interpretation
Farooqi et al. [6]	QUADAS-2 adapted for external validation of commercial AI systems	Independent retrospective clinical cohort; sample size was relatively small	Longitudinal clinical documentation with calibrated examiner adjudication strengthened the reference standard	External validation design reduced concern about overlap with model development, but proprietary training details were unavailable	Code, model architecture, and clinical data were not publicly available because commercial systems were evaluated	Some concern, mainly due to proprietary-system opacity and limited reproducibility resources
Zhu et al. [7]	QUADAS-2/AI-specific appraisal	Multicenter design with one internal and three external centers; external datasets were smaller than the internal set	Expert-established PRG diagnosis and subtype classification were used; clinical reader comparison was included	Internal and external center separation was clear, but detailed leakage-prevention procedures were not fully reproducible from open resources	Code, dataset, and trained model availability were not clearly reported	Low concern for external-validation design, with some concern for reproducibility
Fang et al. [8]	QUADAS-2/AI-specific appraisal	Two-center periapical-radiograph dataset with external testing across acquisition differences	Expert quality grading and technical-error labels were based on prespecified radiographic-quality criteria	Internal and external testing were separated by center/acquisition source	Grad-CAM was reported, but code, dataset, and model availability were not clearly reported	Low concern for external testing, with some concern for open reproducibility
Julião et al. [9]	AI-specific appraisal	External evaluation across multiple CBCT systems and clinical conditions	Refined automatic segmentation and manual segmentation in a subset supported the reference assessment	Multi-device generalizability was explicitly evaluated, although leakage-prevention details were not extensively reported	Evaluated AI software and CBCT data were not openly available	Some concern, mainly due to dependence on non-open software and imaging datasets
Frenkel et al. [10]	QUADAS-2 adapted for image-dataset validation	Independent dental-photograph dataset; patient-level metadata and clinical-source information were limited	Dental-team consensus classification was used	External image independence was clear, but patient-level independence could not be fully verified	AI model was accessible through a web application; code and original training data were not open	Some concern, mainly due to limited patient-level metadata and incomplete reproducibility resources
Azgari et al. [11]	QUADAS-2	Independent patient-level external validation set was used	CBCT-based root-number assessment provided a strong reference standard	Separation of training/validation data and external patient-level testing was described	Source code was publicly available; patient imaging data were restricted for ethical and legal reasons	Low concern for validation design, with residual concern due to restricted data access
Schneider et al. [13]	AI-specific appraisal for federated segmentation	Nine-center international panoramic-radiograph dataset	Tooth segmentation was performed by one expert and verified by a second independent expert	Federated, centralized, and local learning were clearly compared across centers	Multicenter design supported external transportability, but code, data, and trained models were not openly available	Low concern for multicenter validation structure, with some concern for independent reproducibility
Rubak et al. [14]	AI-specific appraisal for federated segmentation and robustness testing	Six-institution distributed dataset; robustness scenarios were experimentally introduced	Tooth segmentation annotations were used, and label inaccuracy was directly modeled	Federated, centralized, and local learning were evaluated under label-noise, image-noise, and faulty-client scenarios	Robustness reporting was strong, but open code, data, and model resources were not clearly available	Low concern for robustness design, with some concern for external reproducibility
Schwarzmaier et al. [24]	QUADAS-2 adapted for external photographic validation	Independent external photograph set; sample size was relatively small and anatomically restricted to anterior deciduous teeth	Visual evaluation by a dental study group was used	External validation of a previously developed model reduced concern about development-test overlap	Freely accessible model was evaluated, but dataset and code were not openly available	Some concern, mainly due to small external sample and limited open resources
Jones et al. [25]	QUADAS-2/AI-specific appraisal	Independent external cohort of carious teeth from children	ICCMS-based practitioner annotation was used	Development and external cohorts were separated, and external performance was reported	Code, scan data, annotations, and trained model were not openly available	Some concern, mainly due to limited external cohort size and restricted reproducibility
Baraka et al. [26]	AI-specific appraisal for CBCT segmentation	Internal and external CBCT datasets were used; the external set was smaller than the internal dataset	Expert-annotated segmentation was used	Internal and external CBCT testing were clearly distinguished	Data and code were available through request-based repositories; public ToothFairy data supported partial reproducibility	Some concern, mainly due to smaller external set and request-based reproducibility resources
Oz et al. [27]	AI-specific appraisal for cross-dataset reproducibility	AKUDENTAL was evaluated against external panoramic-radiograph datasets	Annotation heterogeneity was explicitly examined across datasets	Cross-dataset testing directly evaluated transferability across label protocols	Dataset, documentation, and code were publicly available for non-commercial academic use	Low concern for reproducibility and transparency, with residual concern from label heterogeneity
Aung et al. [28]	AI-specific appraisal for 3D scan segmentation	Public challenge data were complemented by a clinical intraoral-scan validation set	Manual segmentation by experienced clinicians was used	Public-to-clinical generalization was described, although clinical validation was based on a limited number of scans	Public challenge data were available; clinical scans, code, and trained model were not clearly open	Some concern, mainly due to small clinical validation set and incomplete open resources
Chen et al. [29]	QUADAS-2/AI-specific appraisal	Internal dataset plus external open-source image dataset	Internal expert annotation agreement was reported; external label harmonization was less clear	Internal and external datasets were separated	External data were open-source; internal dataset, code, and model were not clearly available	Some concern, mainly due to limited transparency of internal resources and external label comparability
Li et al. [30]	AI-specific appraisal for measurement-model validation	Large internal dataset and independent external tooth-level test set were used within a standardized workflow	Measurements derived from 3D intraoral scans provided a strong reference standard	Internal and external datasets were separated; standardized acquisition reduced uncontrolled variability	Dataset availability was reported through the TDE repository, but code and model availability were unclear	Low concern for validation and reference standard, with some concern for code/model transparency

AI, artificial intelligence; CBCT, cone–beam computed tomography; Grad-CAM, gradient-weighted class activation mapping; ICCMS, International Caries Classification and Management System; PRG, palatal radicular groove; QUADAS-2, Quality Assessment of Diagnostic Accuracy Studies 2; TDE, Tooth Distance Estimation.

**Table 7:** Feasibility assessment for exploratory quantitative synthesis.

Candidate Quantitative Synthesis	Studies Considered	Main Comparability Problem	Decision
External-validation area under the curve	Zhu et al. [7], Fang et al. [8], Frenkel et al. [10], Azgari et al. [11], Schwarzmaier et al. [24]	Although these studies reported external discrimination metrics, the target conditions and tasks differed substantially, including PRG diagnosis, radiographic quality control, root-number detection, and caries detection/classification. Imaging modalities and reference standards were also different.	Not pooled; findings were retained in the structured narrative synthesis.
External-validation Dice similarity coefficient	Julião et al. [9], Schneider et al. [13], Rubak et al. [14], Baraka et al. [26], Aung et al. [28]	Dice values were reported for different segmentation targets and imaging modalities, including posterior teeth in CBCT, teeth in panoramic radiographs, pulp and dental hard tissues in mixed dentition CBCT, and teeth/gingiva in three-dimensional scans.	Not pooled; segmentation performance was interpreted by task and modality.
Internal-to-external performance change	Fang et al. [8], Frenkel et al. [10], Azgari et al. [11], Schwarzmaier et al. [24], Jones et al. [25], Baraka et al. [26], Oz et al. [27], Li et al. [30]	Internal and external performance were reported using different metrics, units of analysis, and validation structures. Confidence intervals or standard errors were not consistently available for paired internal and external estimates.	Not pooled; direction and nature of performance change were summarized qualitatively.
Federated versus centralized or local learning	Schneider et al. [13], Rubak et al. [14]	Only two studies directly evaluated federated learning, and they differed in dataset structure, experimental design, and robustness scenarios.	Not pooled; findings were synthesized narratively within the privacy-preserving learning domain.

CBCT, cone–beam computed tomography; PRG, palatal radicular groove.

**Table 8:** GRADE-informed certainty assessment by functional domain.

Functional Domain	Contributing Studies	Main Evidence Supporting the Judgment	Main Reasons for Downgrading	Overall Certainty
External validation and independent testing	All included studies [6,7,8,9,10,11,13,14,24,25,26,27,28,29,30]	All studies evaluated performance beyond internal model development through external datasets, independent cohorts, multicenter testing, cross-dataset evaluation, or federated/distributed validation. This provides consistent evidence that the final corpus addressed external transportability rather than internal accuracy alone.	External validation designs were heterogeneous, and some external datasets were small or based on image repositories with limited patient-level metadata. Confidence intervals or standard errors were not consistently reported across all external metrics.	Moderate
Multicenter, multi-device, and cross-domain generalizability	Zhu et al. [7], Fang et al. [8], Julião et al. [9], Schneider et al. [13], Rubak et al. [14], Jones et al. [25], Baraka et al. [26], Oz et al. [27], Aung et al. [28], Chen et al. [29], Li et al. [30]	Several studies tested models across centers, devices, acquisition systems, public-to-clinical datasets, or cross-dataset benchmarks. The evidence consistently showed that generalization can be assessed empirically and that domain shift may influence performance.	The studies addressed different imaging modalities and tasks, including CBCT, panoramic radiographs, periapical radiographs, intraoral scans, photographs, and RGB images. Domain-shift mechanisms were not evaluated uniformly, and external performance was not always compared with internal performance using the same metric.	Low to moderate
Cross-dataset reproducibility and annotation heterogeneity	Rubak et al. [14], Frenkel et al. [10], Azgari et al. [11], Baraka et al. [26], Oz et al. [27], Chen et al. [29], Li et al. [30]	The most informative studies directly examined cross-dataset performance, label inconsistency, image noise, or resource availability. AKUDENTAL and Rubak et al. provided particularly relevant evidence that annotation and data-quality differences can affect apparent model performance.	Few studies directly assessed annotation heterogeneity. Open access to code, datasets, or trained models was inconsistent, and many studies could not be independently reproduced from publicly available resources.	Low
Privacy-preserving and federated learning	Schneider et al. [13], Rubak et al. [14]	Both studies empirically compared federated learning with centralized and/or local learning in panoramic-radiograph tooth segmentation. The evidence supports federated learning as a plausible route for multicenter collaboration without direct raw-image sharing.	Only two studies contributed direct evidence. Both focused on panoramic-radiograph segmentation, limiting applicability to other dental imaging modalities and clinical tasks. Performance remained sensitive to data heterogeneity, label noise, and client-level variation.	Low
Transparency and explainability	Fang et al. [8], Frenkel et al. [10], Azgari et al. [11], Schwarzmaier et al. [24], Baraka et al. [26], Oz et al. [27], Li et al. [30]	Some studies included explicit interpretability methods, accessible models, public code, public datasets, or repository-based data availability. These elements improved the ability to understand or partially reproduce model behavior.	Formal explainability methods were uncommon, and most studies did not provide the full combination of code, trained weights, datasets, and detailed preprocessing needed for complete independent reproduction. Reporting of uncertainty was also inconsistent.	Low
Clinical implementation readiness	Farooqi et al. [6], Zhu et al. [7], Fang et al. [8], Julião et al. [9], Jones et al. [25]	A subset of studies moved beyond technical validation by evaluating commercial decision-support systems, clinician-AI comparison, reader assistance, workflow-oriented quality control, segmentation refinement burden, or clinical cohort performance.	Evidence remained indirect for routine implementation. Most studies did not evaluate prospective deployment, real-time integration, patient outcomes, cost-effectiveness, regulatory impact, clinician behavior over time, or post-deployment monitoring.	Very low to low

AI, artificial intelligence; CBCT, cone–beam computed tomography; GRADE, Grading of Recommendations Assessment, Development and Evaluation; RGB, red–green–blue.

## Data Availability

The datasets used and/or analysed during the current study are available from the corresponding author upon reasonable request.

## Supplementary Materials

The following supporting information can be downloaded at: https://www.mdpi.com/article/10.3390/healthcare14131952/s1. Table S1. Search strategies for bibliographic databases. Table S2. Studies excluded after full-text or scope-refinement assessment [5,34,35,36,37,38,39,40,41].

## Abbreviations

The following abbreviations are used in this manuscript:

AUC	area under the curve
AI	artificial intelligence
CBCT	cone–beam computed tomography
CL	centralized learning
DSC	Dice similarity coefficient
FL	federated learning
HD95	95th percentile Hausdorff distance
IoU	intersection over union
LL	local learning
mAP	mean average precision
mIoU	mean intersection over union
NPV	negative predictive value
PRG	palatal radicular groove
SWiM	Synthesis Without Meta-analysis
