# Supplementary material for: From Algorithmic Performance to Clinical Translation: Translational Readiness of Imaging-Based Artificial Intelligence in Dentistry—A Systematic Review
Source: Healthcare (Basel). 2026 Jul 1;14(13):1952. doi: 10.3390/healthcare14131952 (PMC13361231; doi:10.3390/healthcare14131952)
Supplement: Supplementary file 1 [file healthcare-14-01952-s001.zip › Supplementary Table S1 Search Strategies.pdf]

Supplementary Table S1. Search strategies for bibliographic databases

Database	Search strategy
PubMed/MEDLINE	(<br>"artificial intelligence"[tiab] OR "machine learning"[tiab] OR "deep learning"[tiab]<br>OR "neural network*"[tiab] OR "convolutional neural network*"[tiab]<br>OR "federated learning"[tiab] OR "explainable AI"[tiab]<br>OR "explainable artificial intelligence"[tiab] OR XAI[tiab]<br>)<br>AND<br>(<br>dental[tiab] OR dentistry[tiab] OR dentist*[tiab] OR "oral health"[tiab]<br>OR "dental caries"[tiab] OR caries[tiab]<br>OR "dental radiograph*"[tiab] OR "panoramic radiograph*"[tiab]<br>OR bitewing[tiab] OR "periapical radiograph*"[tiab]<br>OR "intraoral scan*"[tiab] OR "intraoral image*"[tiab]<br>OR "dental photograph*"[tiab] OR "tooth segmentation"[tiab]<br>)<br>AND<br>(<br>"external validation"[tiab] OR "externally validated"[tiab]<br>OR "independent validation"[tiab] OR "independent dataset"[tiab]<br>OR "external dataset"[tiab] OR "external cohort"[tiab]<br>OR "independent cohort"[tiab] OR "cross-national validation"[tiab]<br>OR generalizability[tiab] OR generalisability[tiab]<br>OR multicenter[tiab] OR multicentre[tiab]<br>OR "multi-center"[tiab] OR "multi-centre"[tiab]<br>OR "cross-center"[tiab] OR "cross-centre"[tiab]<br>OR "federated learning"[tiab] OR "privacy-preserving"[tiab]<br>)<br>NOT<br>(<br>review[pt] OR systematic review[pt] OR meta-analysis[pt]<br>)
Scopus	TITLE-ABS-KEY (<br>"artificial intelligence" OR "machine learning" OR "deep learning"<br>OR "neural network*" OR "convolutional neural network*"<br>OR "federated learning" OR "explainable artificial intelligence"<br>OR "explainable AI" OR XAI<br>)<br>AND TITLE-ABS-KEY (<br>dental OR dentistry OR dentist* OR "oral health" OR "dental caries" OR caries<br>OR "dental radiograph*" OR "panoramic radiograph*" OR bitewing<br>OR "periapical radiograph*" OR "intraoral scan*" OR "intraoral image*"<br>OR "dental photograph*" OR "tooth segmentation"<br>)<br>AND TITLE-ABS-KEY (<br>"external validation" OR "externally validated" OR "independent validation"<br>OR "independent cohort" OR "independent dataset" OR "external dataset"<br>OR "external cohort" OR "cross-national validation"<br>OR generalizability OR generalisability<br>OR multicenter OR multicentre OR "multi-center" OR "multi-centre"<br>OR "cross-center" OR "cross-centre"<br>OR "dataset shift" OR "domain shift" OR transportability<br>OR reproducibility OR replicability<br>OR "privacy-preserving" OR "data protection" OR "data sharing"<br>OR "federated learning"<br>)<br>AND NOT TITLE-ABS-KEY (<br>"systematic review" OR "meta-analysis" OR "scoping review" OR "narrative review"<br>)
Embase	(<br>'artificial intelligence'/exp OR 'machine learning'/exp OR 'deep learning'/exp<br>OR 'artificial intelligence':ti,ab,kw OR 'machine learning':ti,ab,kw OR 'deep learning':ti,ab,kw

Database	Search strategy
	OR 'neural network*':ti,ab,kw OR 'convolutional neural network*':ti,ab,kw<br>OR 'federated learning':ti,ab,kw OR 'explainable artificial intelligence':ti,ab,kw<br>OR 'explainable AI':ti,ab,kw OR xai:ti,ab,kw<br>)<br>AND<br>(<br>'dentistry'/exp OR 'dental radiography'/exp OR 'cone beam computed tomography'/exp<br>OR dental:ti,ab,kw OR dentistry:ti,ab,kw OR dentist*:ti,ab,kw OR 'oral health':ti,ab,kw<br>OR 'dental caries':ti,ab,kw OR caries:ti,ab,kw<br>OR 'dental radiograph*':ti,ab,kw OR 'panoramic radiograph*':ti,ab,kw<br>OR bitewing:ti,ab,kw OR 'periapical radiograph*':ti,ab,kw<br>OR 'cone beam computed tomography':ti,ab,kw OR cbct:ti,ab,kw<br>OR 'intraoral scan*':ti,ab,kw OR 'intraoral image*':ti,ab,kw<br>OR 'dental photograph*':ti,ab,kw OR 'tooth segmentation':ti,ab,kw<br>)<br>AND<br>(<br>'external validation':ti,ab,kw OR 'externally validated':ti,ab,kw<br>OR 'independent validation':ti,ab,kw OR 'independent dataset':ti,ab,kw<br>OR 'external dataset':ti,ab,kw OR 'external cohort':ti,ab,kw<br>OR 'independent cohort':ti,ab,kw OR 'cross-national validation':ti,ab,kw<br>OR generalizability:ti,ab,kw OR generalisability:ti,ab,kw<br>OR multicenter:ti,ab,kw OR multicentre:ti,ab,kw<br>OR 'multi-center':ti,ab,kw OR 'multi-centre':ti,ab,kw<br>OR 'cross-center':ti,ab,kw OR 'cross-centre':ti,ab,kw<br>OR 'dataset shift':ti,ab,kw OR 'domain shift':ti,ab,kw OR transportability:ti,ab,kw<br>OR reproducibility:ti,ab,kw OR replicability:ti,ab,kw<br>OR 'privacy-preserving':ti,ab,kw OR 'data protection':ti,ab,kw<br>OR 'federated learning':ti,ab,kw<br>)<br>NOT<br>(<br>'review'/it OR 'systematic review'/it OR 'meta analysis'/it OR 'conference abstract'/it<br>)
