# Supplementary material for: From Algorithmic Performance to Clinical Translation: Translational Readiness of Imaging-Based Artificial Intelligence in Dentistry—A Systematic Review
Source: Healthcare (Basel). 2026 Jul 1;14(13):1952. doi: 10.3390/healthcare14131952 (PMC13361231; doi:10.3390/healthcare14131952)
Supplement: Supplementary file 1 [file healthcare-14-01952-s001.zip › Supplementary Table S2 Excluded Studies.pdf]

Supplementary Table S2. Studies excluded after full-text or scope-refinement assessment

These studies were considered during topic development or full-text screening but were not retained in the final strict corpus. The final corpus was restricted to image-based dental AI studies that empirically evaluated external validation, independent image- or patient-level testing, multicenter/multi-device generalizability, cross-dataset reproducibility, or privacy-preserving/federated learning.

Study	Topic / AI task	Reason for exclusion
Szabó et al., 2024	Validation of an AI application for caries detection in intraoral radiographs	The study evaluates reliability/diagnostic assistance of an AI application but was not retained for the strict corpus because the available information did not establish a robust independent external validation, multicenter/multi-device generalization, cross-dataset reproducibility, or privacy-preserving learning component comparable with the final eligibility threshold.
Asiri, 2025	Explainable deep learning models for enamel caries classification in intraoral images	The study contributes explainability, but the final review was restricted to studies with external validation, independent image-dataset testing, cross-dataset reproducibility, multicenter/multi-device generalization, or federated/privacy-preserving learning. A model based only on internal/public-dataset evaluation with illustrative explainability was considered insufficient for the strict corpus.
Mohammad-Rahimi et al., 2024	Self-supervised deep learning for external cervical resorption detection and differentiation from caries	Methodologically interesting for label efficiency/self-supervised learning, but the study was not retained because it did not provide clear external validation, cross-center or cross-device generalization, federated/privacy-preserving learning, or cross-dataset reproducibility.
Schwendicke et al., 2021	Cost-effectiveness of AI for proximal caries detection	Important for implementation economics, but outside the final eligibility focus. The review is centered on empirical external validation, generalizability, privacy-preserving learning, reproducibility, and transportability of image-based AI models rather than economic modeling based on previously reported model performance.
Wu et al., 2025	Predicting plaque-gingivitis risk in schoolchildren using an interpretable machine learning model	Although the study includes a prospectively collected external validation cohort and SHAP-based interpretation, it uses questionnaire and clinical-examination variables rather than dental image data. The final corpus was restricted to image-based dental AI models to preserve conceptual and quantitative coherence.
Chisini et al., 2026	Machine learning models for identifying dental pain in adolescents	The study includes temporal external validation and fairness analysis, but it is based on survey/public-health variables and a self-reported pain outcome rather than dental imaging. It was excluded to maintain the final review focus on image-based dental AI models.
Liu et al., 2026	ToothXpert multimodal large language model for orthopantomography analysis	The study is image-related and includes an external dataset, but it evaluates an interactive multimodal large language model with question-answer outputs rather than a conventional diagnostic, segmentation, or measurement model with directly comparable external-validation metrics. It was excluded to avoid mixing MLLM conversational performance with image-model transportability outcomes.
Mutlu et al., 2026	Deep learning-based automated diagnostic charting on panoramic radiography: YOLOv11 vs YOLOv12	The study used 718 external images from Roboflow Universe to test generalization, but the final strict corpus prioritized studies with clearer patient-level, center-level, device-level, cross-dataset reproducibility, or federated/privacy-preserving validation frameworks. The external set was a public image source incorporated into a hybrid test design, with limited information on patient independence and clinical reference-standard comparability.
Tirkkonen et al., 2026	Explainable and transparent machine-learning model for dental caries prediction using oral-health questionnaire and cohort data	Although the study provides a strong example of cross-national external validation and model explainability, it was excluded because it is not based on dental imaging data. The final corpus was restricted to image-based dental AI models to preserve methodological coherence across imaging modality, reference annotation, validation structure, and potential quantitative synthesis.
